# The nature of multiple boron-nitrogen bonds studied using electron localization function (ELF), electron density (AIM), and natural bond orbital (NBO) methods

**DOI:** 10.1007/s00894-020-04374-9

**Published:** 2020-05-13

**Authors:** Grzegorz Mierzwa, Agnieszka J. Gordon, Slawomir Berski

**Affiliations:** Faculty of Chemistry, University of Wroclaw, 14 F. Joliot-Curie, 50-383 Wroclaw, Poland

**Keywords:** B-N, B=N, B≡N, Chemical bond, Double bond, Triple bond, ELF, Electron localization, Attractor, Basin

## Abstract

Local nature of the boron-nitrogen (BN) bonding with different formal multiplicities (B≡N, B=N, B-N) have been investigated for 25 experimentally established organoboron molecules in both real and the Hilbert space, using topological analysis of electron localization function (ELF), electron density (AIM), and natural bond orbital (NBO) method. Each BN bond has been represented (ELF) by the bonding disynaptic attractor V(B,N), with the basin electron population between 5.72e and 1.83e, confirming possible existence of all the three bond types. A covalent character of bonding can be associated with the dative mechanism due to the V(B,N) bonding basin formed mainly (91–96%) by the N electron density. Similarly, the NBO method shows 2-center natural orbitals, consisting largely of the hybrids from the N atom. The AIM analysis yields the features typical for shared (H_(3,−1)_(*r*) < 0) and closed-shell (∇^2^ρ_(3,−1)_(*r*) > 0) interactions. The delocalization indices, describing electron exchanges between B and N quantum atoms, are smaller than 1.5, even for formally very short triple B≡N bonds.

**Electronic supplementary material:**

The online version of this article (10.1007/s00894-020-04374-9) contains supplementary material, which is available to authorized users.

**Graphical abstract:**
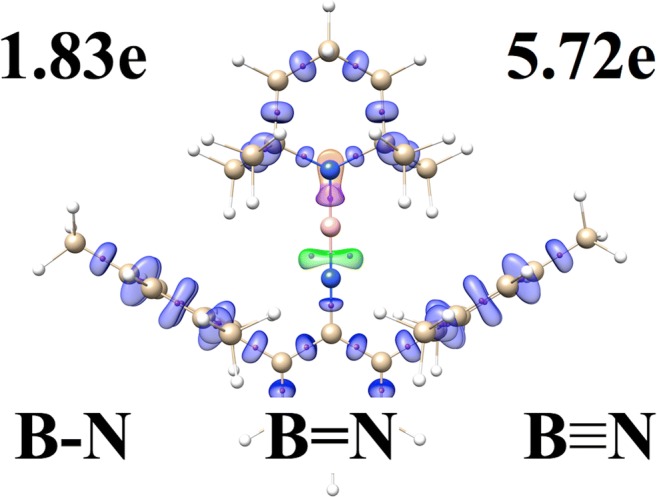
.

## Introduction

The nature of the boron-nitrogen bond (BN) partially determines the physico-chemical properties of molecules, with growing importance in chemical synthesis and applications. Compounds with BN chemical bonds are used to prepare edge-rich, B and N dual-doped graphene sheets (BNGs) to ensure more superior electro-catalytic activity for oxygen reduction [1, 2]. In supramolecular chemistry, scientists can construct crystalline and soft molecular networks using dative BN bonds [3]. The molecular cages containing six dative boron-nitrogen bonds can encapsulate polyaromatic molecules such as triphenylene [4]. Recently, Légaré et al. [5] showed that catenation of two N_2_ molecules (formation of nitrogen chains) under near-ambient conditions is possible using organoboron compound. In the postulated reaction between dinitrogen and a hypothetical base-stabilized borylene, the dipotassium complex is formed with the B^...^N^...^N^...^N^...^N^...^B chain [5]. In an earlier study, Légaré et al. [6] reported that the reaction of N_2_ molecules bound to two borylene units results in the species with the neutral (B_2_N_2_) or dianionic [B_2_N_2_]^2−^ fragments. Such reactions are important for the development of the efficient process of nitrogen fixation and reduction by boron. Zhang et al. [7] demonstrated that the BN bonds, forming the hexagonal boron nitride nanosheet, play an essential role in electrochemical catalysis of N_2_ to NH_3_. It has been postulated that unsaturated boron on the edge can activate inert dinitrogen molecule. In the world of fluorescent organic compounds, incorporation of the B-N bond within the polycyclic aromatic hydrocarbons is a method of rigidifying the compound core, yielding more intense fluorescence, extraordinary thermal and photochemical stability, and high fluorescence quantum yields. For example, Saint-Louis et al. [8] when studying polycyclic azaborine chromophores, designed and synthetized the molecule, where the N-C(=O)-N unit has been replaced with the N-B(OH)-C unit. The obtained azaborine molecules displayed higher molar absorption coefficient than their carbonyl analogs and presented higher emission quantum yields when compared with the imide analogs. In summary, exploration of the BN bond properties is important for the development of new organic field-effect transistors, solid-state lasers, biological imaging, or organic light-emitting diodes.

Discussion of molecular properties is very often related to the assumption that atoms in a molecule are connected by chemical bonds. Thus, their length, type, strength, and applied electronic descriptors, as bond population or the degree of delocalization of electron density (e.g., aromaticity), are crucial for finding correlations between the electronic structure and particular properties. From this point of view, knowledge of the BN bond local electronic structure is essential for predicting its physical properties.

Covalent bonds are usually characterized as single, double, triple (i.e., A-A, A=A, A≡A), or higher order according to the expected electron pairs of polarized electrons, which should be spatially localized between the atomic cores. The Lewis formula [9], introduced before quantum chemistry era, where covalent bonds with shared electron pairs are represented by lines are well accepted multiple bond representation in chemistry. Nowadays, the number of electrons in the chemical bond, localized in space between core regions, can easily be calculated using topological concepts such as attractor, attractor’s basin, and separatrix with molecular distributions of electron localization function (ELF) η(*r*) [10–15]. Thus, the number of bonding electrons, predicted using the Lewis formula or molecular orbital theory, can be verified using modern concepts of quantum chemical topology (QCT) [16].

**Fig. 1:**
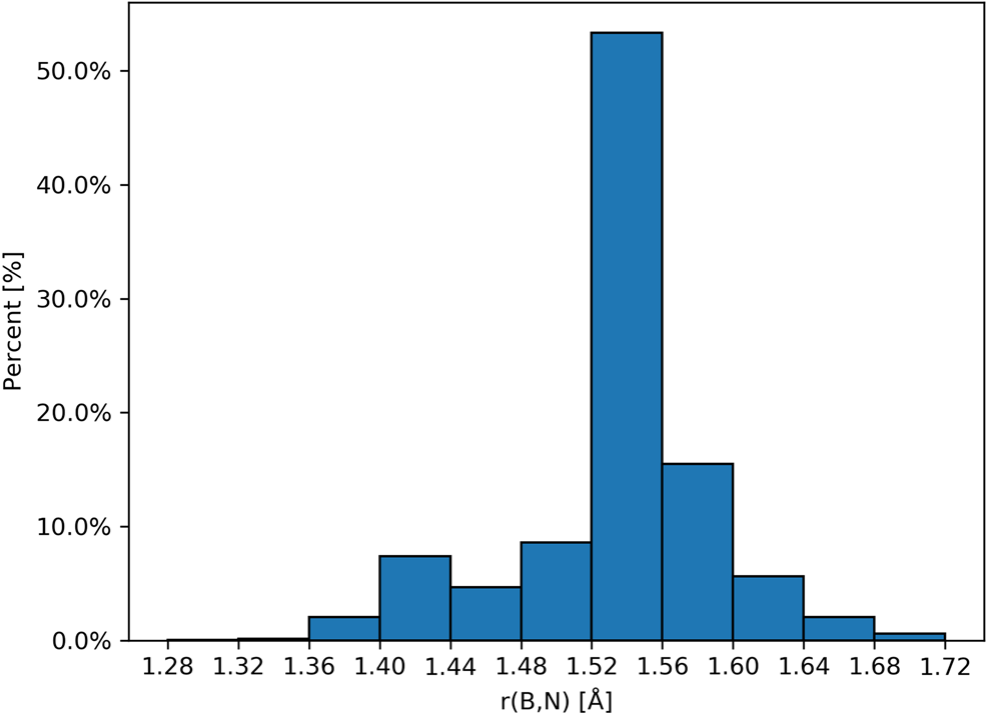
Histogram of distances for the B^...^N contacts in 48,052 crystal structures, obtained from the Cambridge Structural Database

The Cambridge Structural Database (CSD) yielded 48,052 crystal structures with boron-nitrogen contacts between 1.230 and 2.319 Å [17]. Percentage distribution of those contacts is presented in Fig. 1. Two maxima have been observed at approximately 1.43 and 1.54 Å bond lengths, associated with molecules, where the B atom is bonded (mainly) to three and four atoms, respectively.

In the present study, we concentrated on 25 molecules, containing 37 boron-nitrogen bonds selected from the CSD, the prototypical H_3_N-BH_3_ molecule with a single dative bond (N→B), and borazine molecule (B_3_H_6_N_3_) with the delocalized B^...^N bond. Such a variety ensures existence of all the three types of the BN bond, guaranteeing exploration of the nature of the B-N, B=N, and B≡N bonds. The local nature of the boron-nitrogen bonds has been investigated using two methods in the real space, i.e., topological analysis of the electron localization function, η(*r*) (ELF) and topological analysis of electron density, ρ(*r*) (AIM) [18]. Obtained results are independent of a chosen set of molecular orbitals. The third method applied, the natural bond orbital (NBO) method [19], chosen for comparison, is the analysis performed in the Hilbert space.

The main purpose of this study is to describe local electronic structure of the boron-nitrogen bond from the perspective of the topological analysis of η(*r*) and ρ(*r*) fields and to compare the results with those obtained with two-center NBOs. Furthermore, we are interested in verifying to which extent a formal representation of the bond multiplicity (B-N, B=N, B≡N), used in the formal Lewis dot formula [20], stays in agreement with the modern topological analysis of the nature of the BN chemical bond. We also looked for correlations between values characterizing BN bonds, obtained by different approaches and for various bond lengths.

The present study is a part of a larger project, comparing properties of the BB, BC [21], BO [22], BN [23] BF, BCl [24], and BCu [25] bonds, investigated by topological analysis of ELF.

## Computational details

Geometrical structures have been optimized with density functional theory (DFT) method using the Gaussian 09 program (G09) version E.01 [26]. The electron density functionals, i.e., the B3LYP [27–29], M062X [30], ωB97X-D [31], and B97D3 [32, 33] together with the 6-311+G(d,p), 6-311++G(2d,2p) [34–36] and aug-cc-pVTZ [37, 38] basis sets have been used as implemented in G09. For the cojwaa [39] and yecvor [40] molecules, the electrons have been described by the effective core potential (ecp-28) with the def2-TZVP basis set [41] for the Rh and Sn atoms [42] and the 6–311 + G(d,p) basis set for the remaining atoms. The wfn-files for the topological analysis of the η(*r*) and ρ(*r*) fields have been generated using structures optimized at the DFT(M062x)/6-311+G(d,p) computational level for all the molecules, except for cojwaa and yecvor molecules. For those two cases, the all electron basis set TZP [43] without the g-orbitals has been used on the Rh and Sn atoms and the 6-311+G(d,p) basis set on the other atoms in single-point calculations. All optimized structures have been checked for internal/RHF→UHF instabilities.

The def2-TZVP and TZP basis sets have been obtained using the Basis Set Exchange software [44, 45].

The minima on potential energy surface (PES) have been verified through non-imaginary harmonic vibrational frequencies. All the molecules have been studied at 0 K and at singlet electronic states.

The hydrogen atoms have been added to the yecvor molecule, obtained from the CSD before performing the geometry optimization, using the Lewis formula presented in Ref. [40]. Similarly, hydrogen atoms have been added to the cetsup [46], cofvuo [47], bpampb [48] structures, obtained from CSD.

Topological analysis of the ELF has been carried out using the TopMod program with a cubical grid of step size 0.05 bohr [49, 50]. In the case of two or three V_i_(B,N) basins, the simple sum of their basin populations has been considered. In the case of three molecules with the single B-N bond, the monosynaptic non-bonding basin V(N) has been found in the region of the bond instead of the disynaptic basin V(B,N). Such result should be treated with caution because the classification to the synaptic type may depend strongly on numerical details and procedures applied in a program used for topological analysis.

The values of electron delocalization index (DI) in the framework of topological analysis of ρ(*r*) field, have been calculated using the AIMall program [51].

Graphical representations of molecules have been constructed using the JMol program. The ELF domains have been visualized using the VMD [52] and with UCSF Chimera [53], developed by the Resource for Biocomputing, Visualization, and Informatics at the University of California, San Francisco, with support from NIH P41-GM103311.

The NBO analysis has been performed using the version 3.1 of the program, incorporated in the Gaussian 16, Revision E.01 [54]. No “Resonance” and “3CBOND” keywords have been used since only two-center molecular orbitals have been searched for. Analysis has been performed for all optimized molecules at the DFT(M062x)/6-311+G(d,p) computational level.

## Results and discussion

Chemical names of the studied molecules, downloaded from the CSD [17] and the experimental boron-nitrogen bond lengths, *r*_exp_(BN), are presented in Table 1. The molecules contain formal single (B-N), double (B=N), and triple (B ≡ N) bonds and cover the range of *r*_exp_(B,N) values between 1.220 (dogpiy [55]) and 1.717 Å (ajepah [56]).

**Table 1:** The CSD identifier, the experimental BN bond length, *r*_exp_(B,N), and chemical the name of the compound. Chemical names have been obtained from the CSD [17]. For multiple BN bonds, the shortest bond has been presented

CSD identifier	*r*_exp_(B,N) (Å)	Chemical name
dogpiy	1.220	t-Butylnitrido-tris(trimethylsilyl)silyl-borane
vejhib	1.232	t-Butylimino-2,4,6-tri-t-butylphenylborane
sictii	1.254	(2,2,6,6-Tetramethylpiperidin-1-yl)-(2,6-dimesitylphenyliminio)borate
cojwaa	1.255	cis,mer-Dibromo-((trimethylsilyl)iminoboryl)-tris(trimethylphosphine)-rhodium(iii)
cetsup	1.258	t-Butyl-(t-butylimino)-borane
cofvuo^1^	1.364	1,3,5-Tri-t-butyl-2,4,6-tri-isopropyl-3,5-diaza-1-azonia-2,6-dibora-4-borata(2.2.0)bicyclohexane
yecvor^2^	1.377	trans-1,2-bis(Di-isopropylamino-trimethyl-tin-boryl)-ethene
axuviy^3^	1.377	(η6,η6–1,2-bis(Dimethylimino)-1,2-diphenyldiborato)-chromium
zepuo	1.389	9,10-Di-imino-9,10-dihydro-9,10-dibora-anthracene
iditas^4^	1.405	2,4-Bis(dimethylamino)-1,3,5-trimethyl-6-(nitrooxy)borazine
bpampb	1.421	[(4-bromophenyl)(methyl)iminio](chloro)phenylborate
notlud	1.427	10-Hydroxy-6-nitro-10,9-borazarophenanthrene
abitud	1.445	2-ethyl-5-hydroxy-1-methylpyrrolo[1,2-b][2,1]benzazaborinin-3(5H)-one
afucin	1.533	1-(N-(Triphenylphosphonio)anilino)-1-boratabenzene
akesug	1.534	1,1-difluoro-3-phenyl-5-(1H-pyrrol-2-yl)-1H-9,1-pyrrolo[1,2-c][1,3,2]oxazaborepine
amikem	1.558	difluoro-(4-methylaminopent-3-en-2-onato-N,O)-boron
abemez	1.596	trimethylammonio-tricyanoborate
abemid	1.620	trimethylammonio-dicyano(methylmercapto)borate
acipeh	1.630	1-Phenylimidazole-triphenylborane
ajepel	1.705	(2-((2-hydroxyethyl)(benzyl)amino)ethanolato)(fluoro)boron
ajepah	1.717	(2-((2-hydroxyethyl)(methyl)amino)-1,2-diphenylethanolato)(4-methylphenyl)boron

^1^Other bond lengths: 1.565, 1.559, 1.555, 1.549, 1.384, 1.752 Å

^2^Second bond length: 1.410 Å

^3^Second bond length: 1.391 Å

^4^Other bond lengths: ring 1.409, 1.455, 1.442, 1.448, 1.457 Å; the -N(CH_3_)_2_ groups 1.435, 1.431 Å

We have divided selected compounds into three groups, according to the formal BN bonds, published elsewhere. The first group of compounds contains the triple boron-nitrogen bond, B≡N, the second group consists of molecules with formal double bonds, B=N, and the third group gathers compounds with formally single B-N bonds.

All the properties discussed in the text have been obtained for molecules optimized using the DFT(M062X) method and 6-311+G(d,p) basis set, unless stated otherwise.

### The triple B≡N bond

**Fig. 2:**
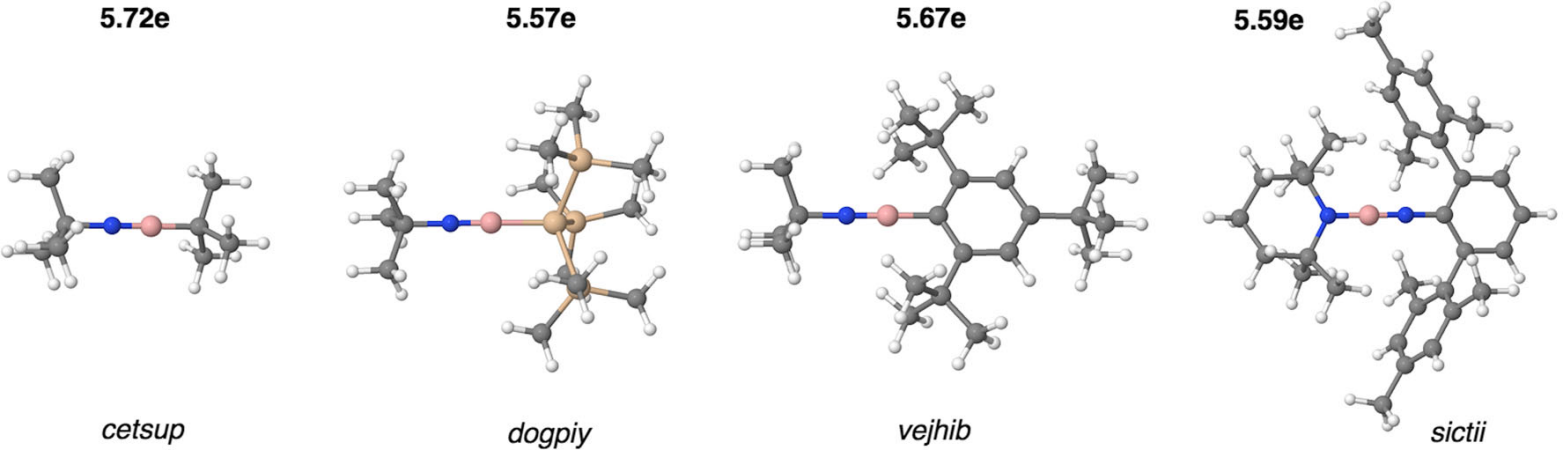
Optimized structures of the cetsup, dogpiy, vejhib, and sictii molecules with postulated triple B≡N bond. Population values for the V(B,N) basins characterizing the BN bonds are shown above

The molecules with formal triple B≡N bond are represented by 4 organoboron molecules, i.e., dogpiy [55], vejhib [57], sictii [58], and cetsup [46], with the *r*_exp_(B,N) bond length between 1.220 and 1.258 Å. The bond length, *r*_opt_(B,N), optimized using the DFT(M062x) method, yielded the values in a very similar range, i.e., 1.241 Å (cetsup)–1.255 Å (sictii). Those values are shown in Table 2, and the bond lengths calculated using the DFT(B3LYP), DFT(B97D3), and DFT(ωB97X-D) methods are shown in Table S1. The optimized structures are shown in Fig. 2.

**Table 2:** Data for the B≡N bond, obtained from topological analysis of electron localization function (ELF) for four molecules with formal triple boron-nitrogen bond. Calculations performed at the DFT(M062x)/6-311+G(d,p) computational level

Mol/aram	r_opt_(B,N) [Å]	$\overline{N}$ [e]	p_NB_	B|V(B,N) [e]	N|V(B,N) [e]	%N
cetsup	1.241	5.72	0.82	0.52	5.19	91
vejhib	1.243	5.67	0.83	0.48	5.19	92
dogpiy	1.245	5.57	0.84	0.44	5.12	92
sictii	1.255	5.59	0.83	0.48	5.09	91

r_opt_(B,N), optimized B≡N bond length; $\overline{N}$, the V(B,N) basin population; _*p*NB_, the polarity index for the V(B,N) basin; B|V(B,N), atomic contributions of the B atom to the V(B,N) basin; N|V(B,N), atomic contributions of the nitrogen atom to the V(B,N) basin; %N, percentage of electron density for the N atom to the V(B,N) basin population

**Fig. 3:**
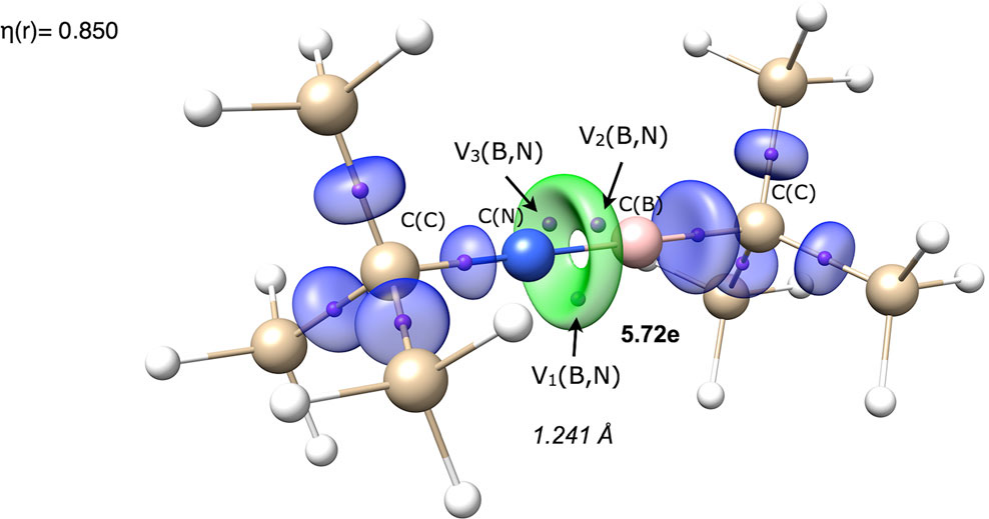
The localization domains (η = 0.850) for the cetsup molecule with basin population values for the B≡N bond. Positions of valence attractors (small violet spheres) have been superimposed on the ball-and-stick representation of the molecular structure. The localization domain in green corresponds to the B≡N bond.

Topological analysis of η(*r*) function shows three (cetsup, dogpiy) or two (sictii, vejhib) bonding disynaptic attractors V_i_(B,N) for the boron-nitrogen bond. According to the interpretation proposed by Silvi and Savin [11], the BN bond has a covalent character with electron density shared by both atoms. The exemplary V_i = 1,2,3_(B,N) attractors in the cetsup molecule are shown in Fig. 3. The V_i = 1,2,3_(B,N) attractors are found outside the imaginary line joining the B and N nuclei. This can be explained by the increased electron localization in the molecular regions with more effective overlapping of the p-orbitals in π-fashion. Similar spatial localization of bonding attractors have been found for the C=C bond [59]. The existence of the three valence V_i = 1,2,3_(B,N) bonding attractors is, however, not an evidence that a single V(B,N) attractor corresponds to any particular (σ or π) bond. The presence of all the three attractors is associated with local symmetry, governed by threefold symmetry axis.

An additional evidence for a triple B≡N bond formation is the absence of the monosynaptic non-bonding attractors V(N), characterizing the non-bonding electron density of the N atom. Since such attractor is not observed, all valence electrons of the nitrogen atom are engaged in the formation of the B≡N and NC bonds. The conclusions above are illustrated well by cetsup. The respective localization domains, corresponding to the CC, BC, B≡N, and NC bonds are presented in Fig. 3.

In order to confirm that the studied short boron-nitrogen bonds are triple bonds, analysis of the basin populations, $\overline{N}$, needs to be performed. Those populations are calculated for localization basins associated with the V_i_(B,N) attractors. The results are presented in Table 2. The total value of $\overline{N}$ ranges between 5.72 (cetsup) and 5.57e (dogpiy). Such large populations undoubtedly indicate the triple B≡N bond. It is worth noting that topological analysis of ELF for the prototypical molecule with the triple C≡C bond, acetylene, HC≣CH studied by Silvi et al. [59] yielded the total population of 5.14e for the V(C,C) basin. Thus, the value of 6e, predicted with the MO or Lewis formula is only a guidance. All bonds with the basin population of V(B,N) larger than 5e in this study are considered to possibly be a triple bond. Since the $\overline{N}$ values are slightly smaller than the formal value of 6e, the resonance equilibrium for structures with the B=N and B≡N bonds should be considered with dominant contribution of the latter.

All the studied B≡N bonds are polar, and the polarization of its electron density is associated with inequivalent character (electronegativity) of the B and N atoms. Polarity can be measured by the polarity index, *p*_NB_. The index has been proposed by Raub and Jansen [60] based on combined topological analysis of ρ(*r*) and η(*r*) fields. It assumes values between 0 for homopolar and 1 for idealized ionic bonds, respectively.

Polarity indices calculated for the B≡N bonds are between 0.82 and 0.84 (see Table 2) and show large polar character of the bonds. Contribution of the electron density from the N atom (5.09e–5.19e) is much larger than that from the B atom (0.44e–0.52e), thus the B≡N bonds have a covalent-polarized character and are strongly polarized towards the nitrogen. Since the atomic contributions of the N atom (91–92%) are very large, it is more appropriate to describe their covalent character with large contribution of the dative mechanism (N→B) than the sharing of electron density by both atoms. Such approach corresponds to formal assumption based on the orbital picture, where electron density from the N atom is donated to empty orbitals of the B atom.

Complementary insight into the bonding nature of the B≡N bonds can be obtained from the topological analysis of electron density, ρ(*r*) [18]. This method is much widely known than the topological analysis of ELF. At this point, it is worth recollecting the two papers by Bader et al. [61, 62] who observed that the fields of the negative Laplacian of ρ(*r*), −∇^2^ρ(r), and ELF are generally homeomorphic [61] and showed that ELF has no direct relationship with the Laplacian of the conditional pair density [62].

The values of electron density for the B≡N bond critical point (BCP), ρ_(3,−1)_(*r*), and other topological parameters are shown in Table 3. As expected, the results are different than those obtained from the ELF representation. The value of the ρ_(3,−1)_(*r*) for four molecules is large (≈ 0.27 e/bohr^3^) and typical for covalent bonds with shared electron density. However, the value of the Laplacian of ρ_(3,−1)_(*r*) for the BCP, ∇^2^ρ_(3,−1)_(*r*), is large and positive (1.19–1.35 e/bohr^5^) indicating a decrease of the electron density around the BCP, typical for closed-shell interactions. It shows that the B≡N bonds cannot be classed as typical covalent-polarized bonds. However, the values of the Cremer and Kraka [63, 64] total energy density, H_(3,−1)_(*r*), are negative (between − 0.27 and − 0.26 au/bohr^3^) and show prevalence of the negative potential energy, typical for the covalent bonds.

**Table 3:** Data for the B≡N bond, obtained from topological analysis of electron density field (AIM) for four molecules with formal triple boron-nitrogen bond. Calculations performed at the DFT(M062x)/6-311+G(d,p) computational level

Mol/param	ρ_(3,−1)_(*r*)	∇^2^ρ_(3,−1)_(*r*)	ε_(3,−1)_	H_(3,−1)_	DI
cetsup	0.271	1.316	< 0.001	− 0.271	1.043
vejhib	0.271	1.310	0.006	− 0.272	0.987
dogpiy	0.268	1.346	< 0.001	− 0.259	1.276
sictii	0.265	1.193	0.059	− 0.266	0.851

The following parameters have been calculated for the B≡N bond critical point, (3,−1): ρ_(3,−1)_(*r*), the value of electron density (e/bohr^3^); ∇^2^ρ_(3,−1)_(*r*), the electron density Laplacian (e/bohr^5^), ε_(3,−1)_, the ellipticity, H_(3,−1)_, the Cremer and Kraka [63, 64] energy density (au/bohr^3^), H(*r*) = G(*r*) + V(*r*). DI, electron delocalization index, the average number of delocalized electrons between quantum atoms B and N [65]

Another interesting measure of a bond character is the electron DI containing information about the average number of electrons shared between quantum atoms. Practical application of the DI in explaining the nature of atomic interactions has been described by Bader et al. [65] in the article entitled “Where To Draw the Line in Defining a Molecular Structure.” In that article, the representation of the bonding (not bonds [66]) in the adduct between HSiCl_3_ and Cp(CO)_2_Mn molecules have been rationalized on the basis of topological analysis of electron density. The values of DI were 0.19 for LiF (the ionic limit), 2.7 for C_2_, and 1.6 for CO molecules. For the B≡N bonds in the sictii, dogpiy, vejhib, and cetsup molecules, the values of DI are between 0.85 and 1.28. Those values are much smaller than 3, expected for an ideal triple bond, and do not confirm the triple B≡N bond existence. Calculations performed at the same computational level for the HC≡CH and N≡N molecules with triple bond yielded the DI of 2.851 and 3.046, respectively. Such results are in agreement with the formal concept of the triple bond in those molecules.

In summary, the results obtained for the B≡N bonds do not confirm a triple covalent bond with 6e shared between the B and N atoms. The boron-nitrogen interaction in the studied molecules shows some similarities with the N→B bond in the H_3_N-BH_3_ molecule, formally understood as covalent-dative. The signs of the Laplacian of ρ_(3,−1)_(*r*) are positive, however the value of ρ_(3,−1)_(*r*) is about two times smaller than those calculated for the B≡N bond. Such small value can indicate a bond with only 2 electrons in the bonding region, much smaller than six electrons found for the B≡N bond. Recently, a dative bond in a series of small molecules has been investigated by Mebs and Beckmann [67], presenting similar topological characteristic of the BN bond. In summary, within the topological analysis of ρ(*r*) field, a formally triple B≡N bond in cetsup, dogpiy, vejhib, and sictii has the topological bond order (AIM) close to 1.

Finally, B≡N bonds have been analyzed in the Hilbert space, via NBOs [68, 69]. This method has been applied to search for 2-center valence natural orbitals, formed by natural atomic hybrids (h_A_) at the B and N atoms. The occupancies of the NBOs, the percentage contribution of the B and N atoms to the NBOs, and sp^λ^ composition of each natural h_A_ are shown in Table 4. The results show that all the studied BN bonds are triple, since three two-center orbitals have been obtained. Their occupations are close to 2e (> 1.91e) and the NBOs are one σ-bond and two π-bonds. The σ-bond is formed by two sp. hybrids overlap, but two π-bonds are formed in a pure fashion by overlapping of the p-orbitals.

**Table 4:** The results of natural bond orbital (NBO) analysis for the boron-nitrogen bonding, formally triple, in four molecules (cetsup, vejhib, dogpiy, sictii) with the B≡N bond and the cojwaa molecule. Calculations performed at the DFT(M062x)/6-311+G(d,p) computational level

Bond		σ-bond					π-bond					π-bond					σ, π, π
Mol/param	*r*_opt_(B,N) [Å]	Ocp	B%	hyb	N%	hyb	Ocp.	B%	hyb	N%	hyb	Ocp	B%	hyb	N%	hyb	ΣOcp
cetsup	1.241	1.987	24	sp^1.34^	76	sp^0.70^	1.969	22	p^1.00^	78	p^1.00^	1.969	22	p^1.00^	78	p^1.00^	5.925
vejhib	1.243	1.984	23	sp^1.32^	77	sp^0.67^	1.964	21	p^1.00^	78	p^1.00^	1.954	22	p^1.00^	78	p^1.00^	5.902
dogpiy	1.245	1.987	23	sp^1.55^	77	sp^0.66^	1.968	23	p^1.00^	77	p^1.00^	1.967	22	p^1.00^	78	p^1.00^	5.922
sictii	1.255	1.984	23	sp^1.14^	77	sp^0.75^	1.961	21	p^1.00^	79	p^1.00^	1.874	17	p^1.00^	83	p^1.00^	5.819
cojwaa	1.269	1.977	19	sp^1.35^	81	sp^0.78^	1.964	17	p^1.00^	83	p^1.00^	1.962	16	p^99.9^	84	sp^58.7^	5.903

*r*_opt_(B,N), optimized BN bond length; Ocp, a number of electrons‚ residing in the natural bond orbital; B%, percentage of NBO electron density, polarized towards the B atom; N%, percentage of NBO electron density, polarized towards the N atom; hyb, natural bonding hybrid constructed from the B and N atoms natural atomic orbitals; ΣOcp, total number of electrons‚ residing in all NBOs

### The double B=N bond

**Scheme 1:**
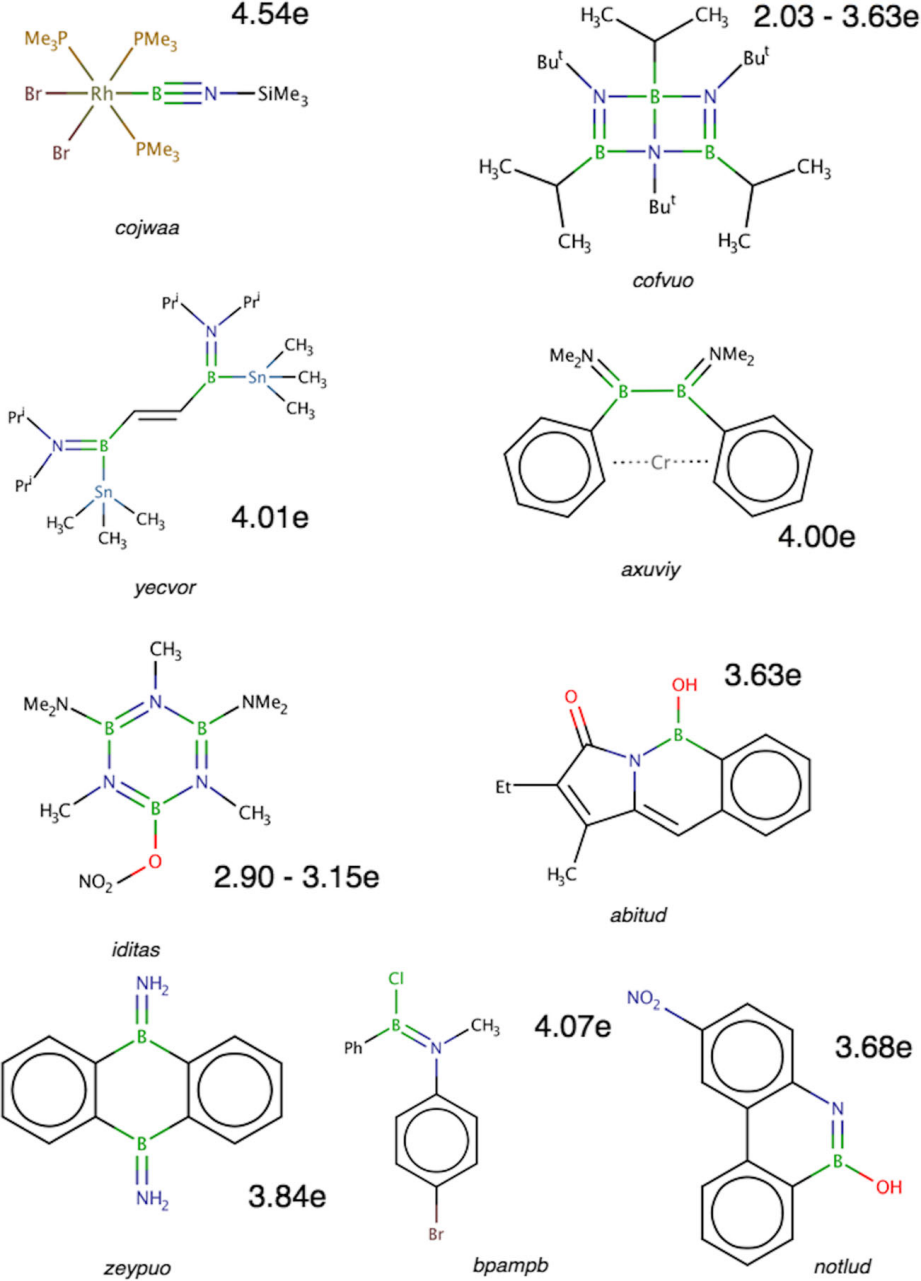
The Lewis formula for organoboron molecules with the double B=N bond. Me, methyl group; Bu^t^, tert-butyl group; Pr^i^, isopropyl group; Ph, phenyl group. The numerical values correspond to the populations of the V(B,N) basins

**Fig. 4:**
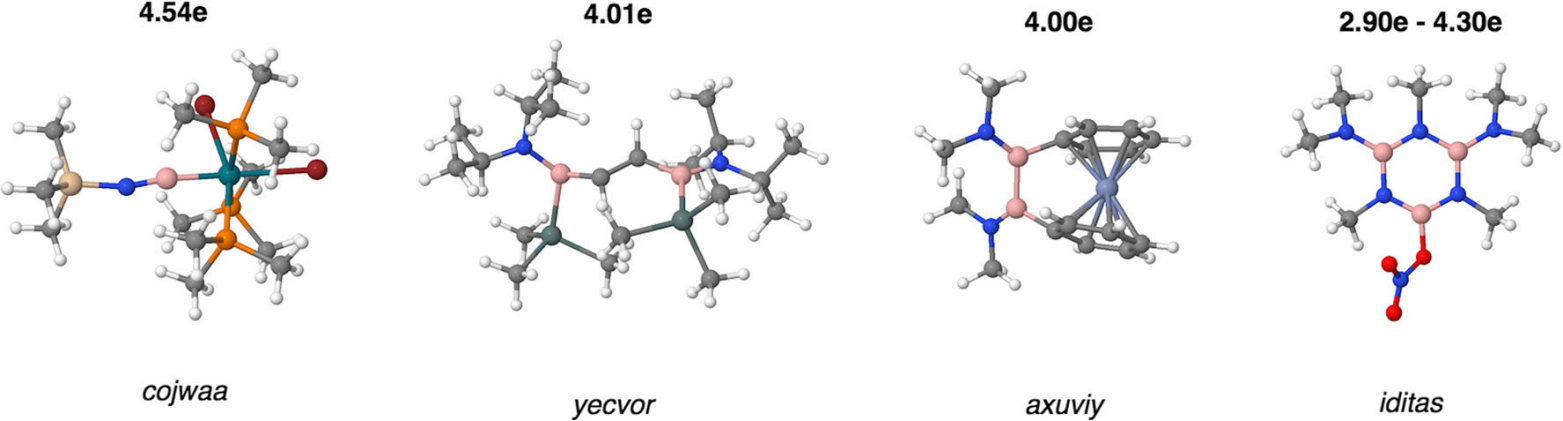
Optimized structures of the cojwaa, yecvor, axuviy, and iditas molecules with postulated double B=N bond. The numerical values correspond to the populations of the V(B,N) basins

In the second part of the research, ten organoboron molecules, i.e., cojwaa [39], cofvuo [47], sictii [58], yecvor [40], axuviy [70], zeypuo [71], bpampb [48], iditas [72], abitud [8], and notlud [73] have been investigated as molecules potentially containing double B=N bonds. Optimized *r*_opt_(B,N) bond lengths are between 1.269 Å in cojwaa and 1.455 Å for one of the BN bonds in the ring of iditas. Experimental *r*_exp_(B,N) values span from 1.255 Å (cojwaa) to 1.457 Å (iditas). Simplified Lewis formulas are shown in Scheme 1, and optimized geometrical structures are presented in Fig. 4.

Each of the studied BN bonds has been characterized by the bonding disynaptic attractor V(B,N), thus the bonds have covalent-polarized character. In the cojwaa, cofvuo, sictii, zeypuo, bpampb, iditas, and notlud molecules, single V(B,N) attractors are observed, while in the yecvor and axuviy molecules, pairs of V_i = 1,2_(B,N) attractors are localized for two BN bonds. Such local topology of ELF is undoubtfully associated with planar geometrical structures of both molecules.

Values of the BN basin populations and other topological parameters obtained from topological analysis of ELF are presented in Table 5. The largest population of 4.54e have been obtained for the shortest (1.255 Å) BN bond investigated in this series of the molecules, i.e., the cojwaa molecule cis,mer-[(Br)_2_(Me_3_P)_3_Rh(B≡NSiMe_3_)]. The BN bond has an interim character between a double (dominant) and a triple bond, since the value of $\overline{N}$ for V(B,N) is larger than a formal value of 4e. Topological analysis of ELF done through localization of the non-bonding attractor and basin V(N) does not show any lone pair on the nitrogen atom. Thus, all valence electron density of the N atom is engaged in formation of bonds with the neighboring atoms, including the NSi bond. This bond, formally single, with the basin population of 3.17e have an interim character between N-Si and N=Si. On the other hand, the B-Rh bond with the basin population of 2.03e perfectly fits the description of the single bond. According to the Braunschweig et al. [39], rhodium iminoboryl complex (cojwaa) has a triple B≡N bond; our analysis, however, suggests the resonance of the two structures, i.e., Rh-B=N^...^Si (dominant) and Rh-B≡N-Si. The polarity index of the BN bond is 0.82, and the BN bond is formed in 91% of electron density from the N atom and 9% from the B atom. Similar to other studied compounds with triple B≡N bonds, the bond is heavily polarized towards the N atom.

**Table 5:** Data for the formal B-N and B=N bonds, obtained from the topological analysis of electron localization function (ELF). Calculations performed at the DFT(M062x)/6-311+G(d,p) computational level

Mol/param	*r*_opt_(B,N) (Å)	$\overline{N}$ (e)	*p*_NB_	B|V(B,N) (e)	N|V(B,N) (e)	%N
cojwaa	1.269	4.54	0.82	0.41	4.12	91
cofvuo^1^	1.384	3.63	0.83	0.30	3.32	92
sictii^2^	1.389	2.88	0.77	0.33	2.55	89
cofvuo^1^	1.390	3.50	0.83	0.29	3.20	92
yecvor ^3^	1.393	4.01	0.88	0.24	3.77	94
axuviy^4^	1.400	4.00	0.90	0.21	3.79	95
zeypuo	1.401	3.84	0.89	0.21	3.63	95
bpampb	1.404	4.07	0.87	0.26	3.80	94
iditas (ring)	1.415	3.15	0.82	0.29	2.85	91
iditas (ring)	1.416	3.05	0.81	0.29	2.76	90
notlud	1.424	3.68	0.88	0.22	3.46	94
borazine	1.429	2.88	0.85	0.22	2.65	92
iditas (B-NMe_2_)	1.435	4.30	0.88	0.25	4.05	94
iditas (B-NMe_2_)	1.436	4.29	0.88	0.25	4.04	94
iditas (ring)	1.449	2.98	0.83	0.25	2.72	92
abitud	1.449	3.63	0.89	0.20	3.42	94
iditas (ring)	1.450	3.06	0.83	0.26	2.80	92
iditas (ring)	1.455	2.99	0.84	0.24	2.74	92
iditas (ring)	1.455	2.90	0.83	0.24	2.65	92

^1)^In the cofvuo molecule, the H-C bonds in two isopropyl groups are oriented in anti positions

^2^The second, longer, BN bond in the sictii molecule

^3^For the yecvor molecule, two V_i = 1,2_(B,N) bonding attractors are localized with the basin populations of 2.00 and 2.01e

^4^For the axuviy molecule two V_i = 1,2_(B,N) bonding attractors are localized with the basin populations of 1.96 and 2.04e

**Fig. 5:**
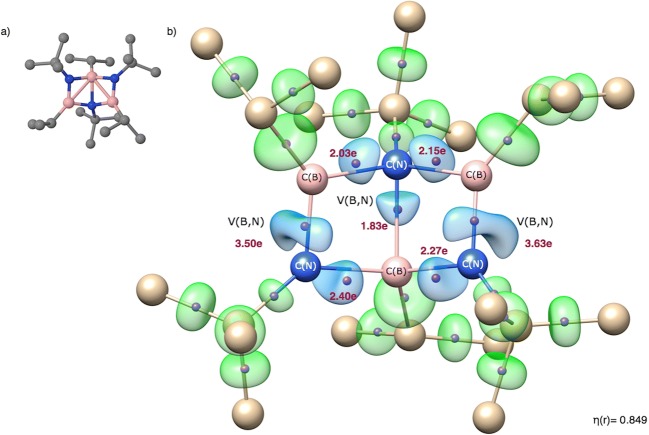
**a** Optimized structure of the cofvuo molecule. Hydrogen atoms have been omitted for clarity. **b** Localization domains (η = 0.849) for the cofvuo molecule. Positions of valence attractors (small violet spheres) have been superimposed on the ball-and-stick representation of the molecular structure. The localization domains in blue corresponds to the BN bonds and in green to other bonds. Localization domains associated with H atoms have been omitted for clarity. The numerical values correspond to the populations of the V(B,N) basins

In the cofvuo molecule, (iPrBNtBu)_3_, two B=N bonds are the shortest of seven B-N bonds and are expected to be of double character (see Scheme 1). The molecule has been synthetized by Paetzold et al. [47] and constitutes an interesting case of six-atom cluster, consisting of three boron and three nitrogen atoms. Its optimized structure is shown in Fig. 5 a. The trimer (iPrBNtBu)_3_ is formed in a process of trimerization of the iPrBNtBu iminoborane [47]. According to Paetzold et al. [47], the molecule has electronic structure resembling borazine with Dewar-type 2-center 2-electron (2c-2e) localized bonds inside the cage. The experimental study [47] showed that peripheral BN bonds are short (1.364 Å, 1.384 Å) and can be characterized as double B=N bonds. The B-N bond inside the cluster is very long (1.752 Å) and most probably a single bond. Four other B-N bond lengths are between 1.549 and 1.565 Å. The values of *r*_opt_(B,N) vary from 1.548 to 1.696 Å. It is worth emphasizing that considered B=N bonds (formally double) are about 0.12 Å longer than the B=N bond analyzed in the cojwaa molecule, thus the topological analysis of ELF should show a smaller double character, interpreted through the values of the basin population. A trivalent character of each boron and nitrogen implies a lone pair on each of the N atoms.

The core and valence attractors of η(*r*) function for the (iPrBNtBu)_3_ molecule are shown in Fig. 5 b and their localization domains in Fig. 5 b. All the boron-carbon, boron-nitrogen, and carbon-carbon bonds are represented by bonding disynaptic attractors V(B,C), V(B,N), and V(C,C) and are in agreement with the Lewis representation of the covalent bonding. Each bonding attractor corresponds to the line between atoms in the Lewis formula and represents a chemical bond. No hypothetical V(N) non-bonding attractors, potentially indicating an existence of a lone pair, are localized near the N atoms. This observation shows that all valence electrons of the N atoms are engaged in formation of BC, BN, and BN covalent bonds, respectively.

Peripheral double bonds, B=N, are characterized by single V(B,N) basins with the basin population of 3.50e (1.390 Å) and 3.63e (1.384 Å). Those values show a prevailing double character of the bonds. However the resonance structures for the B-N and B=N should be taken into consideration in order to correctly describe the nature of the bond with the population smaller than 4e. As predicted, values of $\overline{N}$ for the B=N bonds in cofvuo are 0.91 and 1.04e smaller than the population obtained for the shorter B=N bond in the cojwaa molecule. The B=N bonds are highly polarized with the polarity index *p*_NB_ of 0.83. The contribution of electron density from the N atom is significant (91%).

Formally single B-N bonds are represented by single V(B,N) basins with the populations in the range 1.83–2.40e. The very long B-N bond inside the cage is correctly described by the V(B,N) basin with the smallest population (1.83e). Such depopulated bond, with respect to formal value of 2e, should be characterized as a bond, which nature results from a certain contribution of ionic structures.

**Fig. 6:**
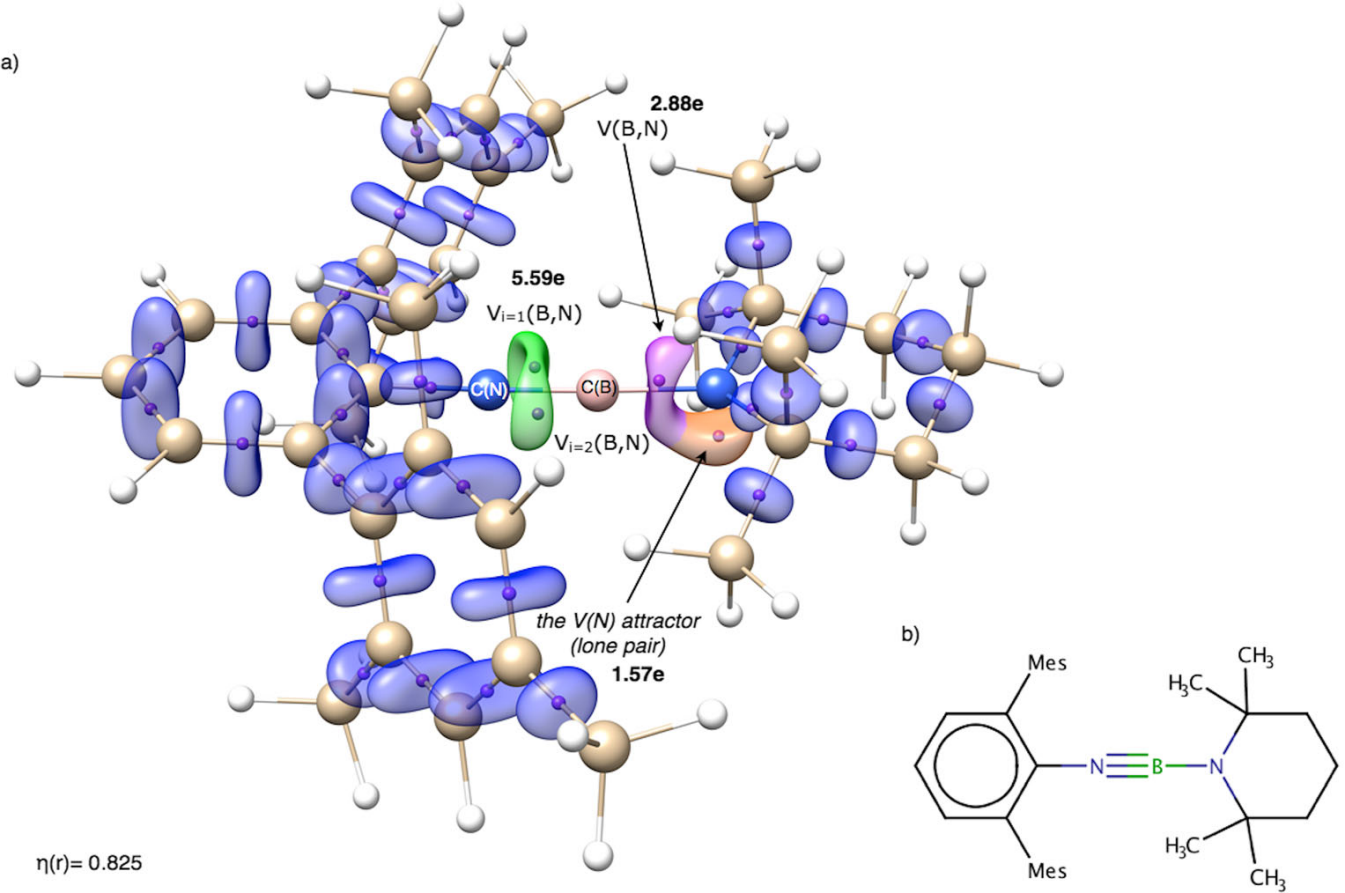
**a** The localization domains (η = 0.825) for the sictii molecule with basin population value for the B=N bond (green). The localization domain for the single B-N is shown in lavender color and the nitrogen lone pair, V(N), is shown in orange. Positions of valence attractors (small violet spheres) have been superimposed on the ball-and-stick representation of the molecular structure. **b** The Lewis formula for the sictii molecule. Mes, mesityl group

The sictii molecule [58] has two boron-nitrogen bonds (see Fig. 2) and its second longer bond (Δ*r*_opt_(B,N) = 0.134 Å) has been considered to have a potentially double character. The shorter BN bond described above has been identified as a triple B≡N bond—this result is in agreement with the interpretation proposed by Rivard et al. [58]. The core and valence attractors in sictii are shown in Fig. 6 a. The optimized B=N bond length is 1.389 Å, a similar value to that obtained for double bonds in the cofvuo molecule (1.384, 1.390 Å). Therefore, a similar value of the $\overline{N}$ for the V(B,N) basin, close to 4e, could be expected. However, the calculated value is 2.88e, much smaller than 3.63 and 3.50e obtained for the BN bonds in the cofvuo molecule. Such result is unexpected, but can easily be explained using the ELF topology. The Lewis formula implies a trivalent N atom, thus two electrons should form a lone pair in its vicinity. Indeed, the monosynaptic non-bonding attractor V(N) is localized (see Fig. 6) near the C(N) core basin. The basin population of V(N) is 1.57e, thus not all valence electrons of the N atom are engaged in the formation of the BN and NC bonds. A relatively small (with respect to 4e) population of the BN bond (2.88e) is caused by separation of the lone pair, therefore not participating in the formation of the dative bond to the B atom. To sum up, topological analysis of ELF partially supports the -N≡B-N(C_2_) representation of the chemical bonds in the sictii molecule with the lone pair at the N atom.

Polarity of the BN bond, measured by the *p*_NB_ index value, is relatively small, 0.77. This is the smallest value calculated for all the BN bonds investigated in this series of the molecules. A total of 89% of the electron density in the V(B,N) basin comes from the N atom.

**Fig. 7:**
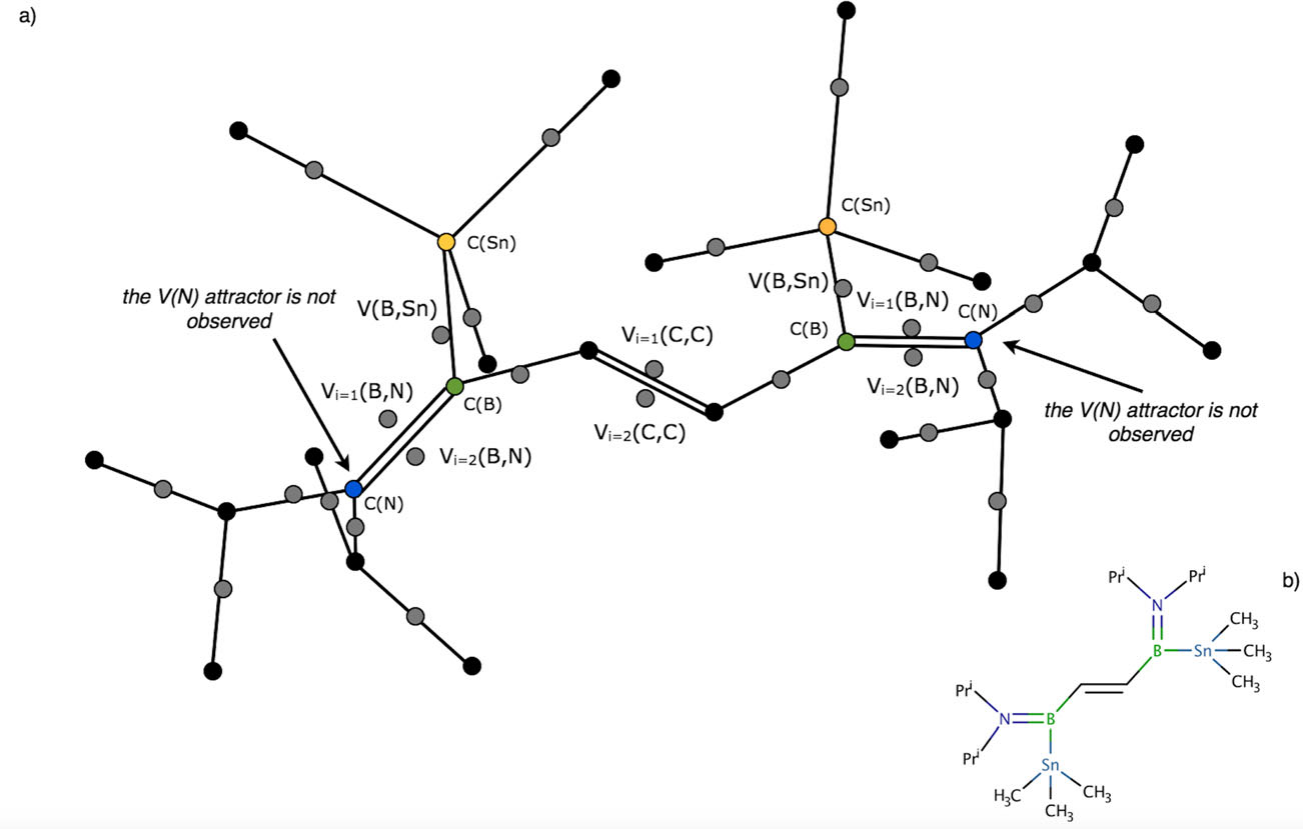
**a** The core and valence attractors in the yecvor molecule. Protonated attractors have been omitted for clarity. b The Lewis formula for the yecvor molecule. Pr^i^, the isopropyl group

The yecvor molecule is an interesting case of the organoboron molecule with two boron-tin (BSn) chemical bonds [40]. Synthesis of yecvor has been described by Frankhauser et al. [40] who characterized the compound using the Lewis formula with two B=N bonds (see Scheme 1). The optimized bond length of both BN bonds (1.393 Å) is only slightly (Δ*r* = 0.003 Å) longer than one of the B=N bonds in the cofvuo molecule, therefore a similar local nature of the bonding is expected. The core and valence attractors of the ELF field are shown in Fig. 7. Two B=N bonds are characterized by the pairs of bonding disynaptic attractors, V_i = 1,2_(B,N). Total population of two V_i = 1,2_(B,N) basins is 4.01e therefore indicating the double B=N bond. Thus, the topological analysis of ELF results confirms the classical interpretation displayed by the Lewis formula. The polarity index, *p*_NB_, of 0.88 is similar to the polarity indices obtained for previously investigated molecules (see Table 5). The results show that the bond is dominated by electron density from the N atom, donating (in total) 3.77e (94%) of the bond’s electron density (basin population).

Particular attention should be paid to the three bonds in yecvor, i.e., the double C=C bond and two, formally single B-Sn bonds. The C=C bond is characterized by two bonding disynaptic attractors V_i = 1,2_(C,C) localized below and above the plane. Such local topology of ELF has been observed for the C_2_H_4_ molecule [12, 59] and is associated with locally planar (approximately) arrangement of two C and two B atoms. However, the total basin population is 3.12e, which is essentially less than 4e expected for the double bond. This can be explained by the resonance forms with the C-C and C=C bonds. Basin populations of two V(B,C), corresponding to the two B-C bonds, formally single, are close to 2e (2.34e), thus the topological analysis of ELF supports the classical picture of the single bond. The BSn bonds are described by single bonding disynaptic attractors, V(B,Sn), with basin populations of 2.45e. The bond is of covalent-polarized type and is formed by 1.34e (55%) from the Sn atom and 1.09e from the B atom. There is a large polarity difference between the B=N bonds with *p*_NB_ of 0.8 and the B-Sn bond with *p*_SnB_ of 0.1.

The second example of the B=N bond, described by a pair of the V_i = 1,2_(B,N) attractors, is the axuviy molecule, [2]borachromoarenophane (Cr{(η^6^-C_6_H_5_)_2_(BNMe_2_)_2_}) [70], in which the Cr atom is situated between two phenyl rings (see Scheme 1). The molecule in singlet electronic state has been investigated. The compound has been described by Braunschweig et al. [70], and the BN bond lengths (1.390, 1.376 Å) have been found within expected double bond range [70]. The *r*_opt_(B,N) value is 1.400 Å. Repeated optimizations carried out using larger basis sets, 6-311++G(2d,2p) and aug-cc-pVTZ, yielded a very similar value of 1.399 Å.

Each N atom in axuviy forms four formally covalent bonds (2xC-N, B=N), therefore the non-bonding electron density (lone pairs) should not be observed in their vicinity. This is confirmed by the topological analysis of η(*r*) function, showing lack of non-bonding V(N) attractors. Thus, the whole valence electron density of the N atom is engaged in formation of chemical bonds. The B=N bonds are described by two pairs of the bonding attractors V_i = 1,2_(B,N). The total population of two V_i = 1,2_(B,N) basins is 4.00e confirming existence of a double B=N bond. The polarity index, *p*_NB_, is 0.89, similar to that of the B=N bonds in the yecvor molecule.

**Scheme 2:**
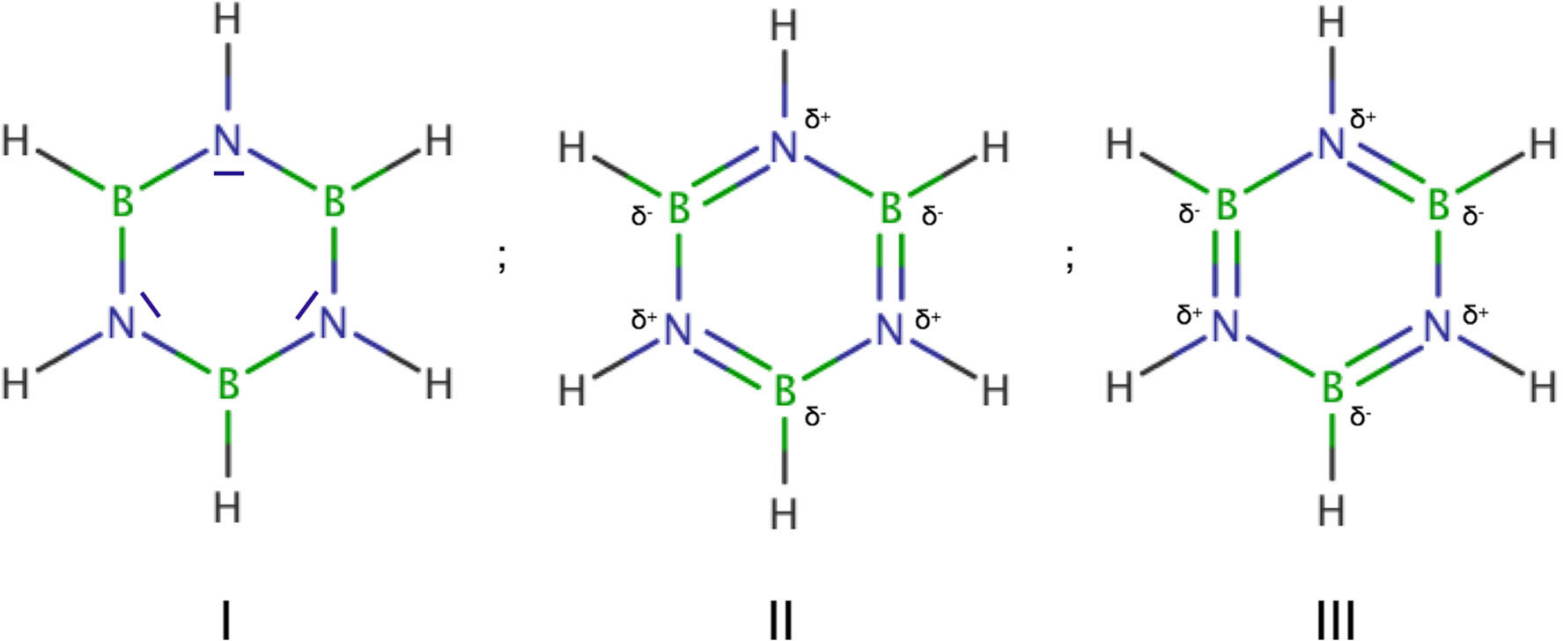
The resonance structures of borazine

Another type of the boron-nitrogen bond has a nature associated with electron delocalization in the atomic ring. Such bonds can be found in the iditas molecule [72] (see Scheme 1). The molecule consists of the borazine ring, B_3_N_3_ with three trimethyl (Me), and two dimethyloamine groups bound to the N and B atoms, respectively. Optimized geometrical structure is shown in Fig. 4 and Fig. S1. A single nitroxy group, -ONO_2_, is bound to the boron atom by the BO bond. The molecule is a borazine derivative, and a set of analogous mesomeric structures can be proposed (see Scheme 2). Two Lewis structures, II and III, suggest the presence of three double B=N bonds and three single B-N bonds in the B_3_N_3_ ring, yielding a formal BN bond order of 1.5. On the other hand, the structure I suggests a single type of the B-N bonding and a lone pair on each the N atom, which should be associated with the V(N) attractor. Optimized bond lengths in the ring (see Fig. S1) are in the range 1.415–1.455 Å. The shortest BN bonds (1.415, 1.416 Å) are localized close to the BONO_2_ molecular fragment. Two *r*_opt_(B,N) bond lengths in the (CH_3_)_2_NB groups are 1.436 and 1.435 Å, those values lie between the shortest and longest bond of the borazine ring.

**Fig. 8:**
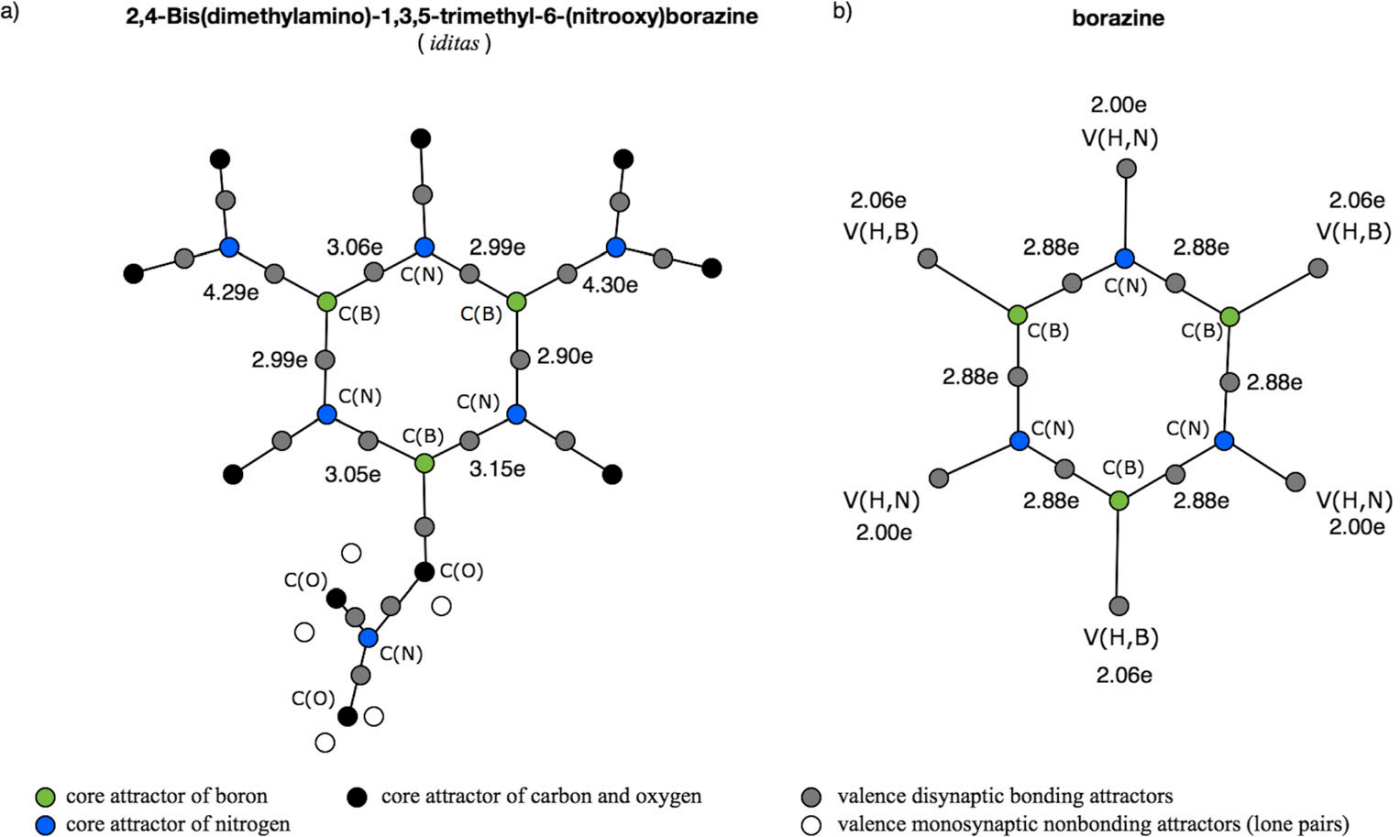
a) The core and valence attractors in the iditas molecule. b) The core and valence attractors in the borazine molecule. Basin populations are shown for selected bonds

The core and valence attractors for the iditas molecule are shown in Fig. 8 a. Topological analysis of ELF shows that six B-N bonds in the borazine ring and two BN bonds of the (CH_3_)_2_NB fragments are described by single attractors and basins V(B,N). No pairs of the disynaptic V_i = 1,2_(B,N) attractors have been found. Such results support delocalized character of the bonding in the ring. For benzene, the prototypical delocalized system, similar single attractors, V(C,C), are observed [11]. In order to find a difference between the iditas and the unsubstituted borazine molecule, topological analysis of η(*r*) function has been performed for borazine. The core and valence attractors for both molecules are compared in Fig. 8 a and b. No differences have been found in the B_3_N_3_ ring ELF topology of both molecules. From ELF topological perspective, the results indicate similar electronic structures for both rings.

Population values for the V(B,N) basins in iditas vary from 2.90 to 4.30e. It is worth emphasizing that the bonds in the B_3_N_3_ ring have smaller population (2.90–3.15e) than single (formal) B-N bonds in the (CH_3_)_2_NB fragments (4.29, 4.30e). Thus, the topological bond order for the bonds in the B_3_N_3_ ring is between 1.45 and 1.56. This result indicates delocalized B^...^N bonds with a formal bond order of 1.5. Each BN bond in borazine has the basin population of 2.88e with the topological bond order of 1.44e—the values are smaller than those obtained for the iditas. The presence of the -NH_2_, -CH_3_, and -ONO_2_ groups in the iditas significantly perturbs the electronic structure of the B_3_N_3_ ring, increasing the population of the BN bonds. Such increase of the basin population can be associated with electron-withdrawing inductive effect of the -ONO_2_ group and electron-releasing inductive effect of the -CH_3_ and -NH_2_ groups [74].

Two BN bonds in the (CH_3_)_2_NB fragments have topological bond order of 2.15 and therefore show a double character. A relatively large amount of electron density localized between the C(B) and C(N) core regions can be associated with the V(N) basin absence (lone pair) on the N atom, and its electron density localized mainly in the boron-nitrogen bond. It is an interesting finding, because the formal Lewis formula does not predict the double character of the N=B bond between the (CH_3_)_2_N group and the B atom of the borazine ring.

For the BN bonds in the iditas borazine ring, the polarity indices are between 0.81 and 0.84. Slightly larger polarities (0.88) have been obtained for the B=N bonds in the (CH_3_)_2_N=B fragments. All the B^...^N bonds in the borazine ring have the same polarity of 0.85, very similar to the value obtained for the iditas. Changes in polarity in the BN bonds due to substitution are very small.

The BN bond in the next three molecules, i.e., zeypuo [71], bpampb [48], and notlud [73] is formally represented by a double B=N bond. The value of *r*_opt_(B,N) is between 1.401 and 1.424 Å, and *r*_exp_(B,N) ranges between 1.389 and 1.427 Å. The topological analysis of η(*r*) function shows single disynaptic bonding attractor V(B,N) for each BN bond. Basin populations for the BN bonds are as follows: 3.84e (zeypuo), 4.07e (bpampb), and 3.68e (notlud), respectively. Thus, for the longest B=N bond in the notlud molecule, the smallest basin population has been obtained. On the other hand, the B=N bond in bpampb, is longer than its equivalent in zeypuo and exhibits a larger value of $\overline{N}$ than that calculated for the shorter bond. The V(N) non-bonding attractor has not been found in any of the three molecules confirming therefore lack of lone pairs on the respective N atoms. Thus, all the valence electron density of N atom is engaged in the formation of covalent bonds. The boron-nitrogen bonds in the studied molecules can be characterized as double B=N bonds, although a single B-N bond, contribution to the mesomeric equilibrium has to be also considered. Like for other investigated molecules, the B=N bonds are formed almost entirely by electron density from the N atom, donating 3.63e (zeypuo) (95%), 3.80e (bpampb) (94%), and 3.46e (notlud) (94%) to the V(B,N) bonding basin.

The last molecule in the series is the abitud molecule. Its structure has been described by Saint-Louis et al. [8] who reported a formal single B-N bond as a part of the six-member ring (see Scheme 1). The molecule contains an unusual N-BOH fragment [8] and intramolecular hydrogen bond, C=O^...^H-O. Furthermore, for the central ring containing the B-N bond, some electron delocalization has been suggested, bound to influence the BN bond properties [8]. Thus, some double character of the bond is likely to be present, and therefore it is worth including the abitud molecule here. The experimental BN bond length of 1.44 Å is very well reproduced by optimization (1.449 Å).

The BN bond in abitud is characterized by the single disynaptic bonding attractor V(B,N). The monosynaptic non-bonding attractor, V(N), has not been found, therefore there is no lone pair on the N atom. Thus, additional electron density—as compared with the formal value of 2e assuming trivalent N atom—should be localized either in the BN bond or in the two NC bonds. Such hypothesis can only be partially confirmed for the BN bond since the value of $\overline{N}$ of V(B,N) is really close to 4e (3.63e). The BN bond has an interim character between a double B=N and single B-N with a larger participation of a double character in the mesomeric equilibrium. The basin populations of 1.96 and 2.11e for the N-C bonds, indicate single bonds, as predicted by the Lewis formula. The polarity index, *p*_NB_, is 0.89, and the bond is formed by 0.20e from the B atom (5%) and 3.42e from the N atom (94%).

Numerical parameters, obtained from topological analysis of ρ(*r*) field, characterizing B=N bonds in the cojwaa, cofvuo, sictii, yecvor, axuviy, zeypuo, bpampb, iditas, abitud, and notlud molecules, are collected in Table 6. Each bond has been described by the BCP. Values of ρ_(3,−1)_(*r*) for the B=N bonds are in the range between 0.267 and 0.185 e/bohr^3^, are generally smaller than those for the triple B≡N bonds (0.271–0.268 e/bohr^3^), and decrease with the bond elongation. Similar decrease in value has been found for the ∇^2^ρ_(3,−1)_(*r*), H_(3,−1)_(*r*), and DI parameters. All the B=N bonds are characterized by positive values of the ∇^2^ρ_(3,−1)_(*r*) in the range from 1.063 to 0.400 e/bohr^5^, smaller than those for the triple B≡N bonds (1.316–1.193 e/bohr^5^). No pattern has been observed for the ellipticity parameter with exceptionally large values (0.156, 0.158) for the sictii and iditas molecules. Total energy density, H_(3,−1)_(*r*), is negative for all the studied B=N bonds, thus the energetics of the bonds (BCP) is dominated by the potential energy density, similarly to the B≡N bonds. Delocalization indices are between 1.018 (cojwaa) and 0.420 (iditas) and do not conform to the concept of a double bond, but suggest bond multiplicity smaller than 1.

**Table 6:** Data for the formal B-N and B=N bonds, obtained from the topological analysis of electron density field (AIM). Calculations performed at the DFT(M062x)/6-311+G(d,p) computational level

Mol/param	*r*_opt_(B,N) (Å)	ρ_(3,−1)_(*r*)	∇^2^ρ_(3,−1)_(*r*)	ε_(3,−1)_	H_(3,−1)_	DI
cojwaa	1.269	0.267	1.063	0.016	− 0.271	1.018
cofvuo	1.384	0.216	0.580	0.069	− 0.208	0.596
sictii	1.389	0.221	0.389	0.156	− 0.225	0.586
cofvuo	1.390	0.211	0.564	0.074	− 0.201	0.599
yecvor	1.393	0.205	0.631	0.022	− 0.186	0.740
axuviy	1.400	0.199	0.654	0.043	− 0.174	0.682
zeypuo	1.401	0.201	0.611	0.067	− 0.179	0.554
bpampb	1.404	0.203	0.554	0.067	−0.186	0.570
iditas (ring)	1.415	0.207	0.426	0.078	− 0.200	0.489
iditas (ring)	1.416	0.207	0.422	0.075	− 0.200	0.492
notlud	1.424	0.194	0.533	0.028	− 0.174	0.455
borazine	1.429	0.195	0.474	0.077	− 0.177	0.506
iditas (B-NMe_2_)	1.435	0.192	0.452	0.158	− 0.173	0.452
iditas (B-NMe_2_)	1.436	0.192	0.449	0.158	− 0.173	0.451
iditas (ring)	1.449	0.189	0.402	0.036	− 0.173	0.457
abitud	1.449	0.182	0.500	0.105	− 0.157	0.425
iditas (ring)	1.450	0.189	0.400	0.040	− 0.172	0.456
iditas (ring)	1.455	0.184	0.414	0.028	− 0.165	0.420
iditas (ring)	1.455	0.185	0.411	0.024	− 0.166	0.443

*r*_opt_(B,N), optimized B-N and B=N bond length; the following parameters have been calculated for the BN bond critical point, (3,−1): ρ_(3,−1)_(*r*), the value of electron density (e/bohr^3^), ∇^2^ρ_(3,−1)_(*r*), the electron density Laplacian (e/bohr^5^), ε_(3,-1)_, the ellipticity; H_(3,−1)_, the Cremer and Kraka [63, 64] energy density (au/bohr^3^); H(*r*) = G(*r*) + V(*r*). DI, electron delocalization index, the average number of delocalized electrons between quantum atoms B and N [65]

The local nature study of the B=N bonds, carried out with the NBO analysis yields the σ and π orbitals (the double B=N bonds) for the cofvuo, yecvor, and iditas molecules. For other molecules, single B-N bonds have been found, accompanied by a lone pair on the N atom, formed by the p-orbital. The occupancy of the NBOs, the percentage contribution of the B and N atoms to the NBOs, and sp^λ^ composition of each natural atomic hybrids h_A_ are shown in Table 7. Double B=N bonds consist of two NBOs formed mainly by the N atoms with atomic contributions of 76–82%. The results support the finding from the topological analysis of ELF, showing that the B=N bonds are formed mainly by the electron density from the N atom.

**Table 7:** The results of natural bond orbital (NBO) analysis for the boron-nitrogen bonding for molecules with formal B-N and B=N bonds. Calculations performed at the DFT(M062x)/6-311+G(d,p) computational level

Bond		σ-bond					π-bond/lone pair				
Mol/param	*r*_opt_(B,N) [Å]	Ocp	B%	hyb	N%	hyb	Ocp	B%	hyb	N%	hyb
cofvuo	1.384	1.955	18	sp^2.03^	82	sp^1.65^	1.918	15	p^99.9^	85	p^99.9^
sictii	1.389	1.976	23	sp^0.88^	77	sp^1.88^	1.779	-	-	98	sp^47.15^
cofvuo	1.390	1.956	19	sp^2.03^	81	sp^1.64^	1.923	15	p^99.9^	85	p^99.9^
yecvor	1.393	1.974	21	sp^2.29^	79	sp^1.23^	1.939	15	p^99.9^	85	p^99.9^
axuviy	1.400	1.979	21	sp^2.43^	79	sp^1.23^	1.650	-	-	100	p^99.9^
zeypuo	1.401	1.988	24	sp^2.31^	76	sp^1.01^	1.734	-	-	100	p^99.9^
bpampb	1.404	1.974	22	sp^2.06^	78	sp^1.35^	1.677	-	-	100	p^99.9^
iditas (ring)	1.415	1.973	23	sp^1.71^	77	sp^1.81^	-	-	-	-	-
iditas (ring)	1.416	1.972	23	sp^1.75^	77	sp^1.79^	-	-	-	-	-
notlud	1.424	1.981	24	sp^2.29^	76	sp^1.42^	1.656	-	-	100	p^99.9^
iditas (B-NMe_2_)	1.435	1.978	23	sp^1.99^	77	sp^1.37^	-	-	-	-	-
iditas (B-NMe_2_)	1.436	1.978	23	sp^1.99^	77	sp^1.37^	1.918	9	p^99.9^	91	p^99.9^
iditas (ring)	1.449	1.970	23	sp^1.97^	77	sp^1.71^	-	-	-	-	-
abitud	1.449	1.979	22	sp^2.25^	78	sp^1.55^	1.619	-	-	100	p^1.00^
iditas (ring)	1.450	1.968	23	sp^1.99^	77	sp^1.72^	-	-	-	-	-
iditas (ring)	1.455	1.967	22	sp^2.05^	78	sp^1.69^	1.803	9	p^99.9^	91	p^99.9^
iditas (ring)	1.455	1.964	22	sp^2.02^	78	sp^1.71^	-	-	-	-	-

### The single B-N bonds

Finally, the B-N bond has been investigated. Formally, such 2-center bond is described by 2e and labeled as a single bond. The H_3_N-BH_3_ molecule has been chosen as a reference system. The molecule also serves as a dative bond prototype. The topological analysis of η(*r*) function, performed using the DFT(M062x) method for the 6-311+G(d,p) and aug-cc-pVTZ basis sets, yields very similar results. In the B-N bonding region, the monosynaptic V(N) basin is observed, supporting a dative-covalent bond, with the nitrogen lone pair donated to the boron atom. It is worth emphasizing that the topological analysis of ELF does not yield any disynaptic basin V(B,N); only monosynaptic V(N) basin is observed. The basin population of V(N) is 1.88e with the 6-311+G(d,p) basis set and 1.91e with the aug-cc-pVTZ basis set, very close to a formal value of 2e. The V(N) basin is formed by 1.80e (1.83e) from the N atom and 0.08e from the B atom, with a very high polarity index of 0.91. Thus, 96% of electron density in the B-N bond region comes from the N atom. It is evident that the topological approach, based on the ELF, applied to the B-N chemical bond in H_3_N-BH_3_ confirms the classical concept of the dative bond N→B.

The NBO analysis gives some more insights. The two-center natural orbital B-N with the population of 1.993e is composed of 19% contribution from the B atom and 81% from the N atom. The bond is formed by overlapping of the natural hybrids, i.e., sp^5.18^ at the B atom and sp^1.76^ at the N atom.

**Scheme 3:**
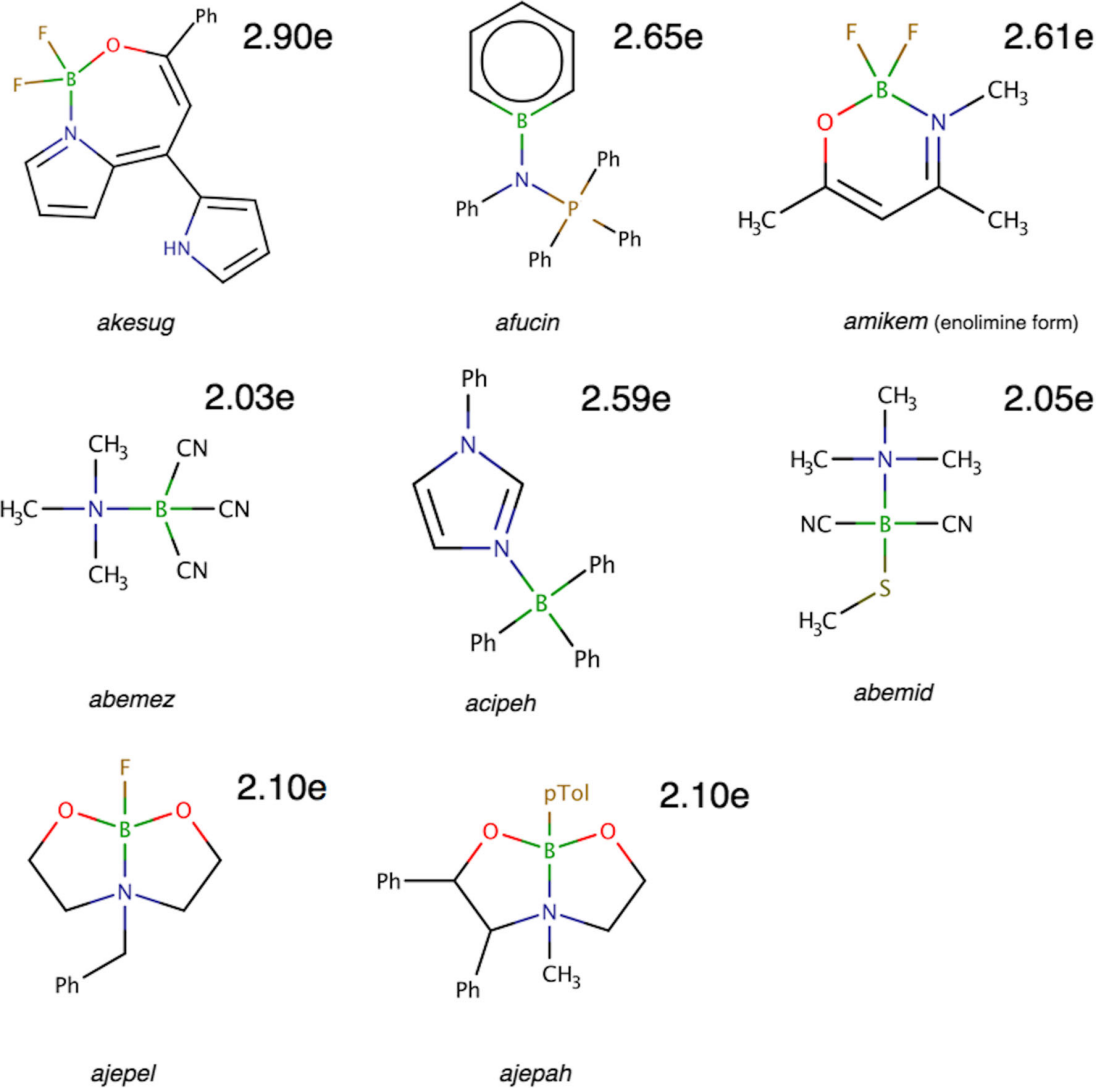
The Lewis formula for organoboron molecules with the single B-N bond. pTol, 4-methylphenyl (p-tolyl) group; Ph, phenyl group. The numerical values correspond to the populations of the V(B,N) basins

It is interesting to find whether the dative N→B bonding, characterized by larger than 95% contribution of electron density from the N atom, can also be found in more complex molecular structures. Therefore, 14 formally single B-N bonds in 10 molecules, with the *r*_opt_(B,N) lengths between 1.547 and 1.785 Å have been investigated. The molecules, selected from the CSD, are as follows: akesug [75], cofvuo, afucin [76], amikem [77], abemez [78], acipeh [79], abemid [78], ajepel [56], and ajepah [56]. The Lewis structures for all the studied molecules are shown in Scheme 3.

Each studied B-N bond is represented by a single valence basin V(B,N) or V(N). Such diversity of topological types is observed only for relatively long bonds, formally considered single. It is evident that all studied B-N bonds are either dative or have some covalent character due to shared electron density. The topology of η(*r*) function, similar to that in the H_3_N-BH_3_ molecule, has been observed in the afucin and acipeh molecules. In the B-N bond region, instead of the expected V(B,N) basin, a single V(N) basin has been localized.

The values of $\overline{N}$ for V(B,N) are collected in Table 8. They range between 2.90e for the akesug molecule with the shortest B-N bond (1.547 Å) and 1.83e for one of the B-N bonds in the cofvuo molecule with the longest B-N bond (1.752 Å). The bonds can be classed as single, although a contribution of double B=N bond should also be considered in a resonance equilibrium (akesug, amikem, acipeh).

**Table 8:** Data for the boron-nitrogen bonds, obtained from the topological analysis of electron localisation function (ELF) for molecules with formal B-N bonds. Calculations performed at the DFT(M062x)/6-311+G(d,p) computational level

Mol/param	*r*_opt_(B,N) (Å)	$\overline{N}$ (e)	*p*_NB_	B|V(B,N) (e)	N|V(B,N) (e)	%N
akesug	1.547	2.90	0.88	0.18	2.72	94
cofvuo	1.549	2.03	0.82	0.18	1.84	91
afucin	1.551	2.65	0.91	0.12	2.53	95
cofvuo	1.555	2.15	0.82	0.19	1.96	91
cofvuo	1.559	2.27	0.84	0.18	2.09	92
cofvuo	1.565	2.40	0.85	0.18	2.22	93
amikem	1.568	2.61	0.83	0.18	2.43	93
abemez	1.629	2.03	0.86	0.14	1.89	93
acipeh	1.632	2.59	0.93	0.10	2.49	96
abemid	1.654	2.05	0.87	0.13	1.91	94
NH_3_-BH_3_	1.655	1.88	0.93	0.07	1.80	96
cofvuo	1.752	1.83	0.86	0.13	1.69	93
ajepel	1.781	2.10	0.86	0.15	1.95	93
ajepah	1.785	2.10	0.88	0.13	1.97	94

**Fig. 9:**
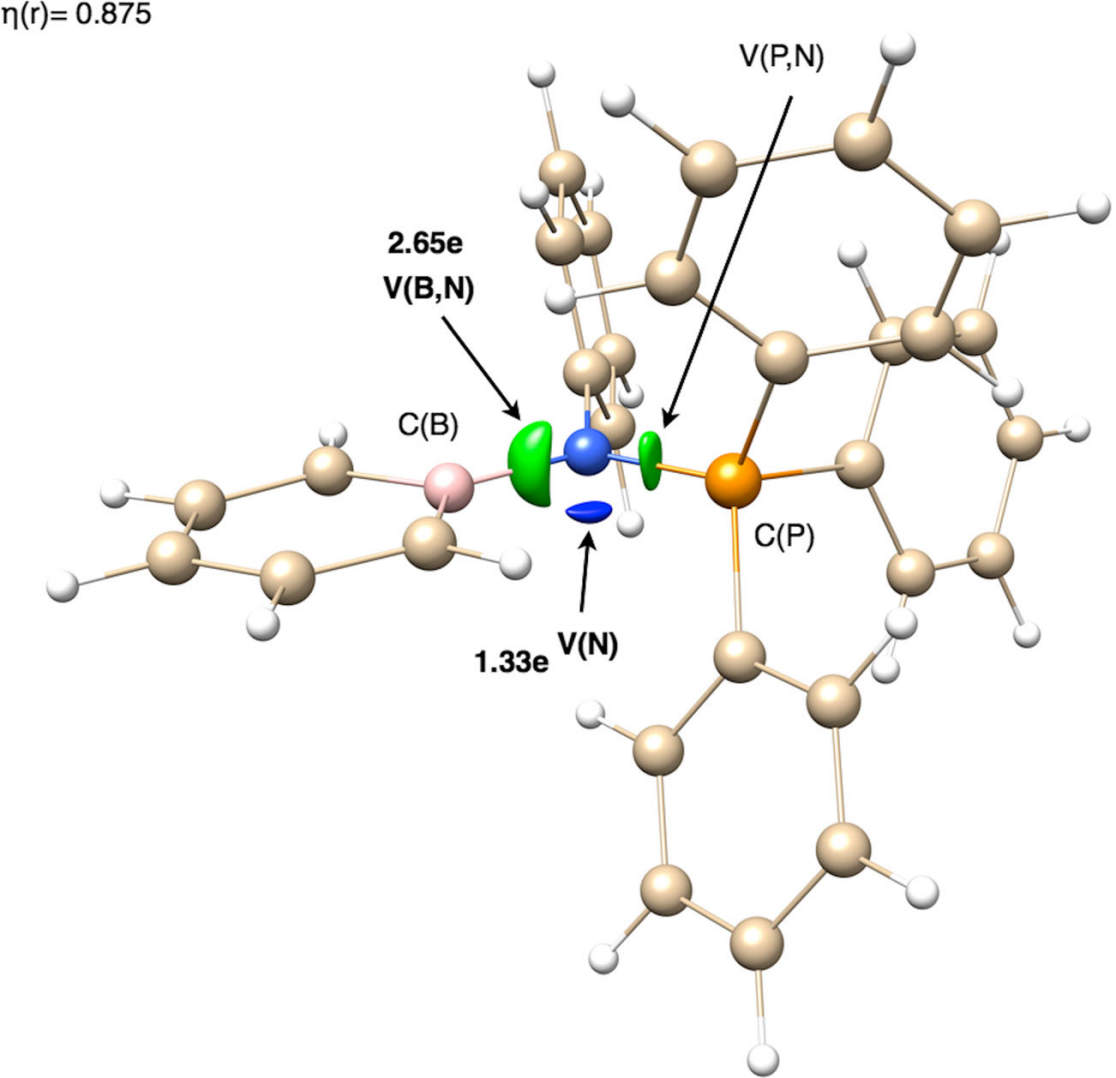
The localization domains (η = 0.875) for the afucin molecule with the BN, PN, and N lone pair basin population marked. The localization domains have been superimposed on the ball-and-stick representation of the molecular structure

More detailed analysis of ELF topology has not shown any lone pair on the N atom for any of the molecules except for afucin, where the monosynaptic non-bonding basic V(N) has been found. The basin population of V(N) in afucin is 1.33e (see Fig. 9). This value is clearly smaller than a formal value of 2e. The remainder of the electron density is found mainly in the BN bond (2.65e). Thus, small contribution of the double boron-nitrogen bonding should be also considered. Nevertheless, the localization of separated V(N) shows that such contribution is rather small.

Polarity indices, *p*_NB_, (see Table 8) are between 0.82 (cofvuo) and 0.93 (acipeh, H_3_N-BH_3_). Their values confirm that for all the studied molecules, the B-N bond is mainly formed by electron density from the N atom, donating from 1.69e (cofvuo) to 2.72e (akesug) to the bond. Such atomic contribution is much larger than that obtained for the B atom in the range from 0.13 (afucin) to 0.19e (cofvuo). Electron density from the N atom contributes from 91 (cofvuo) to 96% (acipeh) to a single B-N. For the prototypical H_3_N-BH_3_ molecule, the percentage contribution of electron density from the N atom is 96%.

**Fig. 10:**
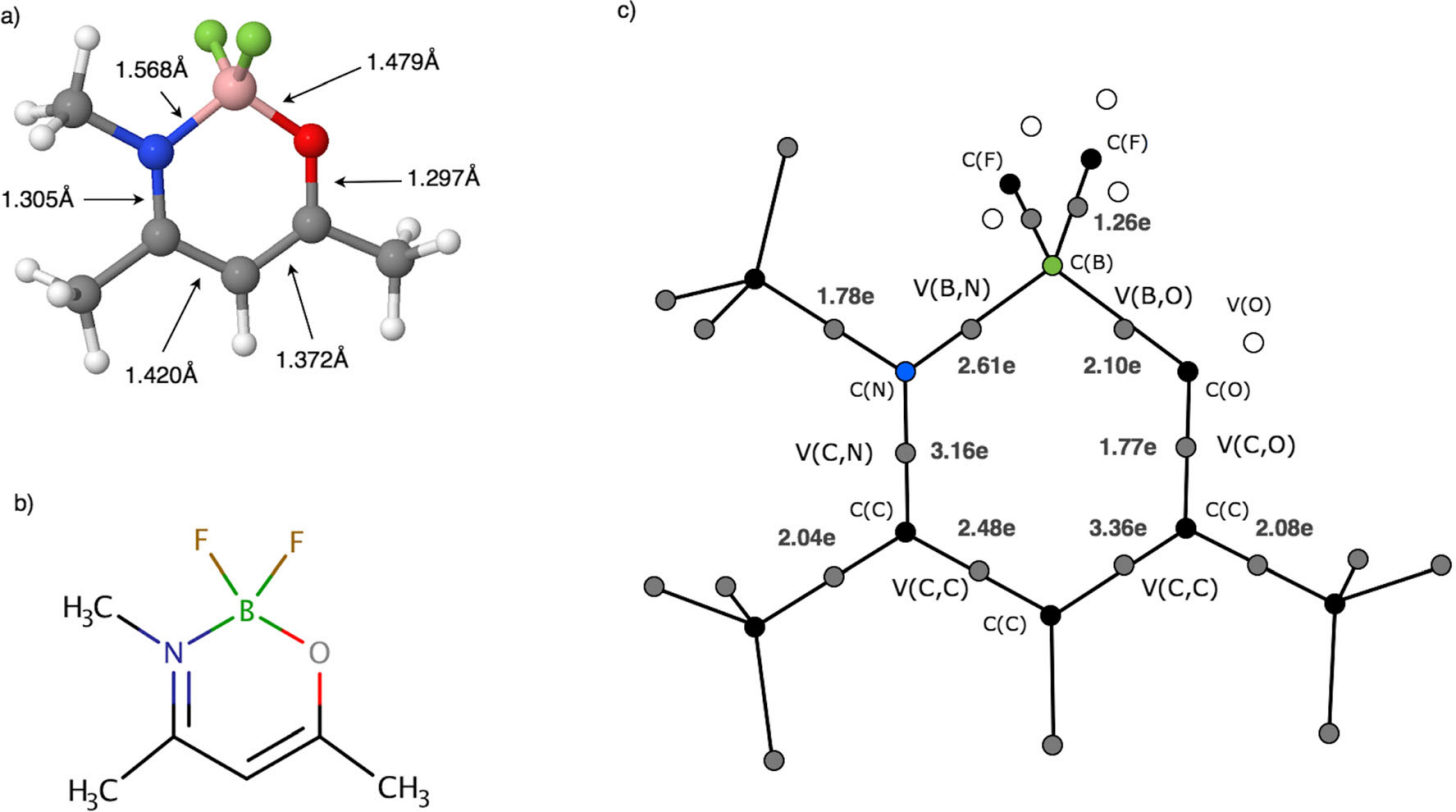
**a** Optimized structure of the amikem molecule. **b** The Lewis formula for the amikem molecule. **c** The core and valence attractors in the amikem molecule. Black circles represent core attractors, gray circles represent valence attractors, blue circle represents C(N), green circle represents C(B), with basin population values for selected bonds

The single B-N bond will be discussed using an exemplar molecule amikem (difluoro-(4-methylaminopent-3-en-2-onato-N,O)-boron). This compound has been reported by Itoh et al. [77] as a complex between BF_2_ and olefin. Its optimized structure is shown in Fig. 10 a. The X-ray structure of the molecule has been discussed in the context of a possible mixture of ketoamine and enolinine tautomers [77]. In the ketoamine form, the double C=O bond is present, while in the enolimine form (see Fig. 10b) the C=N bond is observed. Each of the forms, or their weighted average, should be confirmed by the ELF results. Topological analysis of η(*r*) function shows 37 attractors, including 11 core attractors and 26 valence attractors. All attractors are shown in Fig. 10 c. Each covalent bond, predicted by the Lewis formula, is represented by the bonding disynaptic attractor. For example, the BN bond is characterized by the V(B,N) attractor, the BO bond by the V(B,O) attractor and two BF bonds by the V(B,F) attractors. Boron-fluorine bonds have partially covalent character and were studied in detail by us before [24]. In the valence shell of the N atom, the non-bonding V(N) attractor is not observed, thus whole valence electron density is localized in the CN bond and B-N bonds. This is partially supported by basin population values: the CN bond of the ring is 3.16e, the BN bond, formally single, has 2.61e, but the C-N bond formed by the methyl group has 1.78e. The double C=C bond, expected in the enolimine resonance form, has the population of 3.36e. The values of $\overline{N}$ for all bonds are presented in Fig. 10 c. Comparison of basin populations with formal numbers of electrons for the bonds clearly shows that more than one Lewis structure has to be used to describe chemical bonding in the amikem. Nevertheless, the enolimine form is partially confirmed due to the populations of the V(C,N) and V(C,C) basins, which are 3.16 and 3.36e (close to the formal value of 4e), representing double C=N and C=C bonds respectively.

Finally, single B-N bonds are investigated using the topological analysis of ρ(*r*) field. It shows the BCP for every B-N bond investigated in this series. The numerical parameters characterizing BCPs are shown in Table 9. The electron densities for the BCPs are in the range from 0.150 (akesug, cofvuo) to 0.093 e/bohr^3^ (ajepah), related to the values of *r*_opt_(B,N) from the shortest to the longest. Values of ∇^2^ρ_(3,−1)_(*r*) for the BCP are positive (0.461–0.068 e/bohr^5^). The largest value (0.461 e/bohr^5^) has been obtained for the prototypical H_3_N-BH_3_ molecule. Interpretation of the Laplacian sign is similar to those obtained for the B≡N and B=N bonds: electron density is removed around the BCP as in the case of the prototypical H_3_N-BH_3_ molecule. For two other parameters, i.e., the ellipticity of ρ_(3,−1)_(*r*) and total energy density for the BCP, only the values of H_(3,−1)_(*r*) show a decrease along with elongation of *r*_opt_(B,N) bond length. Those values are between − 0.122 au/bohr^3^ (akesug, cofvuo) and − 0.056 au/bohr^3^ (H_3_N-BH_3_). The most interesting parameter, the DI, related to the multiplicity of the bond, does not show any essential dependence from the *r*_opt_(B,N) bond length. Its value changes from 0.215 (ajepel) to 0.402 (afucin). The number of delocalized electron pairs is less than 1, expected for a single bond, and it oscillates around 0.336 for the delocalization of electron density between the B and N quantum atoms in the H_3_N-BH_3_ molecule.

**Table 9:** Data for the boron-nitrogen bonds, obtained from the topological analysis of electron density field (AIM) for molecules with formal B-N bonds. Calculations performed at the DFT(M062x)/6-311+G(d,p) computational level

Mol/param	*r*_opt_(B,N) (Å)	ρ_(3,−1)_(*r*)	∇^2^ρ_(3,−1)_(*r*)	ε_(3,−1)_	H_(3,−1)_	DI
akesug	1.547	0.150	0.310	0.021	− 0.122	0.282
cofvuo	1.549	0.147	0.278	0.338	− 0.122	0.376
afucin	1.551	0.132	0.459	0.247	− 0.091	0.402
cofvuo	1.555	0.150	0.302	0.275	− 0.124	0.385
cofvuo	1.559	0.143	0.243	0.158	− 0.118	0.364
cofvuo	1.565	0.143	0.262	0.156	− 0.116	0.364
amikem	1.568	0.144	0.294	0.045	− 0.116	0.278
abemez	1.629	0.129	0.252	< 0.001	−0.100	0.335
acipeh	1.632	0.109	0.428	0.035	− 0.067	0.306
abemid	1.654	0.121	0.228	<0.011	− 0.092	0.348
NH_3_-BH_3_	1.655	0.100	0.461	0.000	− 0.056	0.336
cofvuo	1.752	0.108	0.182	0.074	− 0.081	0.289
ajepel	1.781	0.096	0.068	0.312	− 0.072	0.215
ajepah	1.785	0.093	0.092	0.222	− 0.067	0.239

*r*_opt_(B,N), optimized B-N bond length; the following parameters have been calculated for the B-N bond critical point, (3,−1): ρ_(3,−1)_(*r*), the value of electron density (e/bohr^3^); ∇^2^ρ_(3,−1)_(*r*), the electron density Laplacian (e/bohr^5^); ε_(3,−1)_, the ellipticity; H_(3,−1)_, the Cremer and Kraka [63, 64] energy density (au/bohr^3^); H(*r*) = G(*r*) + V(*r*). DI, electron delocalization index, the average number of delocalized electrons between quantum atoms B and N [65]

The NBO analysis used to find 2-center natural orbitals revealed the B-N bonding only for eight investigated molecules (see Table 10). For the three molecules (akesug, ajepel, ajepah), the standard procedure used has not shown any natural bond orbital between the B and N atoms (see the “Computational details” section). Occupations of NBOs, the percentage atomic contributions, and natural hybrids of atomic orbitals forming the NBOs are presented in Table 10. All identified NBOs have the population close to 2e (1.910e–1.993e). Contribution of the B atom ranges between 15 (cofvuo) and 21% (abemez), and is 5.6 and 3.8 times smaller than the contribution of the N atom. Similar to the B≡N and B=N bonds, single B-N bonds are polarized towards the more electronegative N atom.

**Table 10:** The results of natural bond orbital (NBO) analysis for the boron-nitrogen bonding for molecules with formal B-N bonds. Analysis of the results shows the presence of 2-center NBO between the B and N atoms. Calculations performed at the DFT(M062x)/6-311+G(d,p) computational level

Mol/param	*r*_opt_(B,N) (Å)	Ocp	B%	hyb	N%	hyb
cofvuo	1.549	1.927	17	sp^2.45^	84	sp^2.93^
afucin	1.551	1.957	20	sp^2.91^	80	sp^1.48^
cofvuo	1.555	1.937	17	sp^2.49^	83	sp^2.62^
cofvuo	1.559	1.947	19	sp^2.98^	81	sp^2.12^
cofvuo	1.565	1.947	19	sp^3.10^	82	sp^2.07^
amikem	1.568	1.972	20	sp^3.07^	80	sp^2.12^
abemez	1.629	1.954	21	sp^4.36^	79	sp^2.53^
acipeh	1.632	1.962	18	sp^4.83^	82	sp^1.52^
abemid	1.654	1.956	19	sp^4.17^	81	sp^2.42^
NH_3_-BH_3_	1.655	1.993	19	sp^5.18^	81	sp^1.76^
cofvuo	1.752	1.910	15	sp^4.60^	85	sp^3.89^

**Fig. 11:**
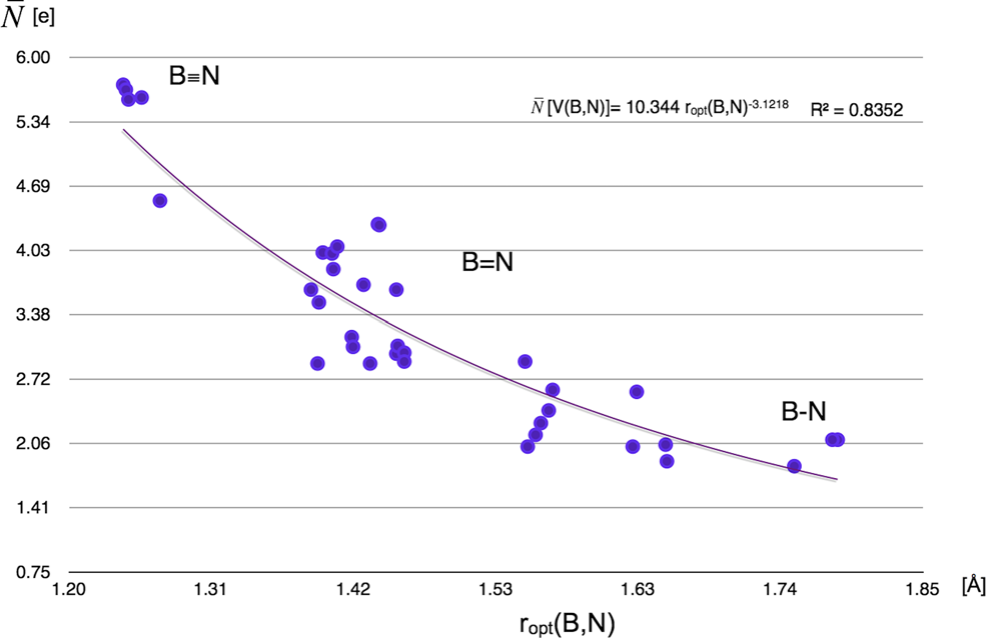
Correlation between basin populations of the BN bonds, $\overline{N}$, obtained from topological analysis of ELF and optimized bond length *r*_opt_(B,N) demonstrated using power regression

In the final part of our study, relationships for the BN local nature parameters, different bond lengths, and formal multiplicity have been analyzed for all the investigated molecules. As has been shown above, the basin populations of the BN bonds support the formal concept of the B-N, B=N, and B≡N bonds. Furthermore, the values are decreasing from 5.72e for the cetsup molecule to 1.83e for the cofvuo molecule along with elongation of the *r*_opt_(B,N). The dependency between the $\overline{N}$ values for V(B,N) and *r*_opt_(B,N), investigated using the regression analysis with the power model, is shown in Fig. 11.

**Fig. 12:**
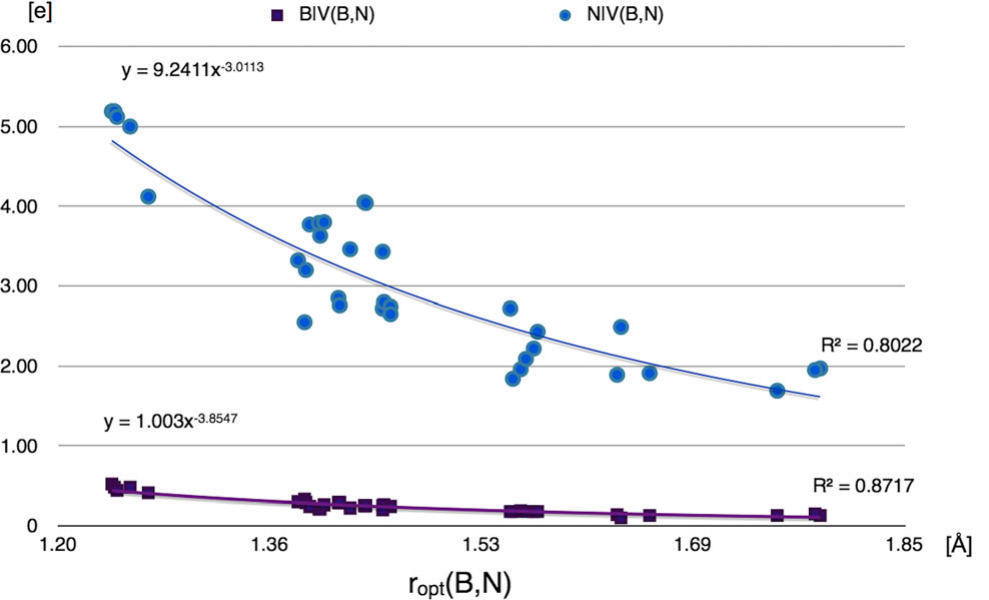
Correlation between electron density contributions of the quantum atoms B and N to bonding basin V(B,N) population and the optimized bond length *r*_opt_(B,N), modeled by power regression. The vertical axis shows atomic contributions

All the studied BN bonds are highly polarized with the polarity indices, *p*_NB_, between 0.77 (sictii) and 0.93 (acipeh, NH_3_-BH_3_). Unfortunately, we have been unable to find any essential correlation between the *p*_NB_ values and the *r*_opt_(B,N) bond lengths (see Fig. S2). The bonds are polarized towards the N atom, which delivers from 5.19e (cetsup, vejhib) to 1.69e (cofvuo) to the bonding V(B,N) basin. The atomic contribution of the B atom ranges between 0.52 (cetsup) and 0.13e (cofvuo), and is much smaller than that of the nitrogen. The correlation between atomic contribution, B|V(B,N) and N|V(B,N) and the *r*_opt_(B,N) has been analyzed with the power regression model, and is presented in Fig. 12. Elongation of the BN bond causes a decrease in the atomic contribution from the N atom, but very small decrease in the atomic contribution from the B atom.

**Fig. 13:**
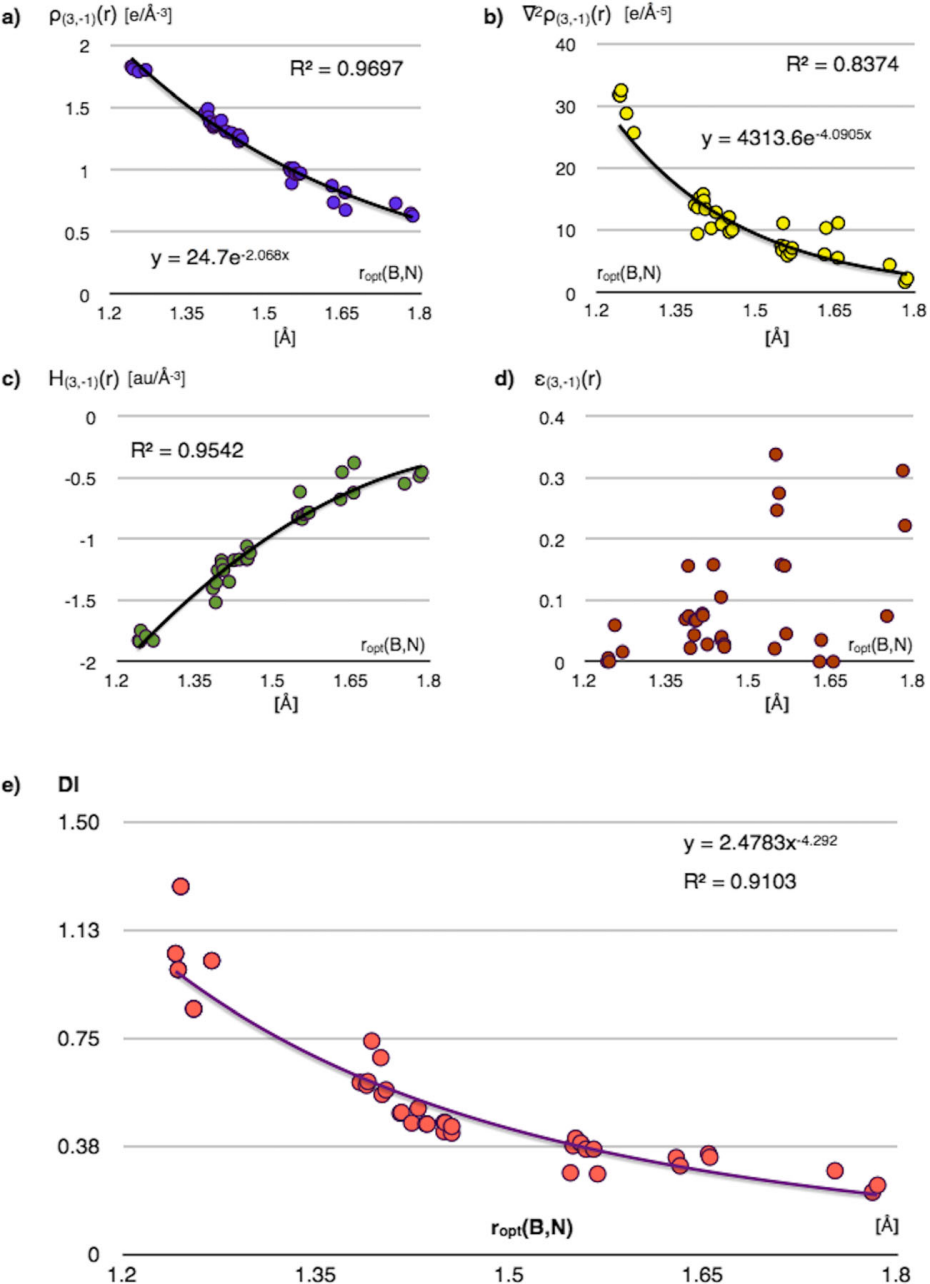
**a** Correlation between electron density value for the (3,−1) BN critical point (CP), ρ_(3,−1)_(*r*), and optimized bond length *r*_opt_(B,N). **b** Correlation between electron density Laplacian for the (3,−1) BN CP, ∇^2^ρ_(3,−1)_(*r*), and *r*_opt_(B,N). **c** Correlation between total energy density for the (3,−1) BN CP, H_(3,−1)_(*r*), and *r*_opt_(B,N), 2nd degree polynomial in H_(3,−1)_(*r*) was used. **d** The ellipticity values for the (3,−1) BN CP, ε_(3,−1)_(*r*), as function of *r*_opt_(B,N). **e** Correlation between the delocalization index, DI, for the B and N quantum atoms, obtained from topological analysis of ρ(*r*) field, and *r*_*opt*_(B,N) demonstrated using power regression

The correlations between the ρ_(3,−1)_(*r*), ∇^2^ρ_(3,−1)_(*r*), H_(3,−1)_(*r*) and ellipticity, ε_(3,−1)_ calculated for the BCPs of the BN bonds, and the value of the *r*_opt_(B,N) bond lengths are shown in Fig. 13 a–d. The regression analysis shows a very good exponential correlation for the ρ_(3,−1)_(*r*) value, good exponential correlation for ∇^2^ρ_(3,−1)_(*r*) and a polynomial correlation for H_(3,−1)_(*r*) values, and no significant dependence for the ellipticity parameter. The values of ε_(3,−1)_ seem to be independent from the BN bond length. The values of the ρ_(3,−1)_(*r*), ∇^2^ρ_(3,−1)_(*r*), and |H_(3,−1)_| decrease along with elongation of the BN bond. The BN bonds, which have partially covalent character with some electron density shared between atoms, show decrease of electron density for the BCP along with the bond elongation.

**Fig. 14:**
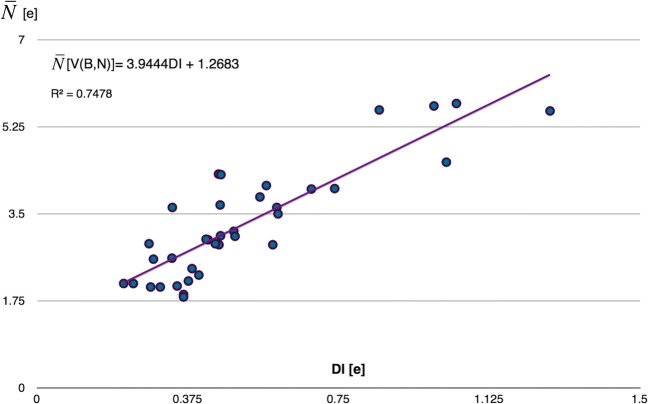
Correlation between the BN bond population, $\overline{N}$, represented in topological analysis of η(*r*) field by the bonding disynaptic basin V(B,N) and the delocalization index, DI, for the B and N quantum atoms in topological analysis of ρ(*r*) field, demonstrated using linear regression

The DI measuring the amount of electron density exchanged between quantum atoms exhibits also some dependence on the *r*_opt_(B,N) value. The power regression analysis is shown in Fig. 13 e. The largest values of the DI are obtained for formally triple B≡N bonds and the smallest ones for the longest bonds, characterized as single B-N. It is worth emphasizing that even for triple bonds, a number of electrons exchanged between the B and N atoms do not exceed 1.28. As shown by Bader et al. [65], the delocalization index provides a physical measure of a property associated with covalence in classical models of bonding. Since the topological analysis of ELF also provides similar measure through localization of the bonding basin V(B,N) and its basin population, $\overline{N}$, therefore it is interesting to compare both approaches. The linear regression has been used to describe the relationship between the DI values for the BN bonds and corresponding values of the basin population for V(B,N). The results are presented in Fig. 14. As can be expected, large DI values correspond to basin population characteristics for triple bonds (5.59e–5.72e), and both parameters decrease with elongation of the BN bonds. Interestingly, when applying the ($\overline{N}$[V(B,N)] = 3.9444DI + 1.2683) formula, the basin population of 1.27e is obtained for DI = 0, when the interaction between the B and N quantum atoms is not covalent. Population of 1.27e can be related to the lone pair V(N), a remainder of the bonding basin V(B,N) at long B^...^N distances. Krokidis et al. [80] showed that dissociation of the N→B dative bond in the prototypical NH_3_-BH_3_ molecule results in the change of the disynaptic bonding V(B,N) basin into the monosynaptic basin V(N). Such topological description corresponds to the classical explanation of a coordination bond. Population of 1.27e can indeed characterize such a V(N) non-bonding basin.

**Fig. 15:**
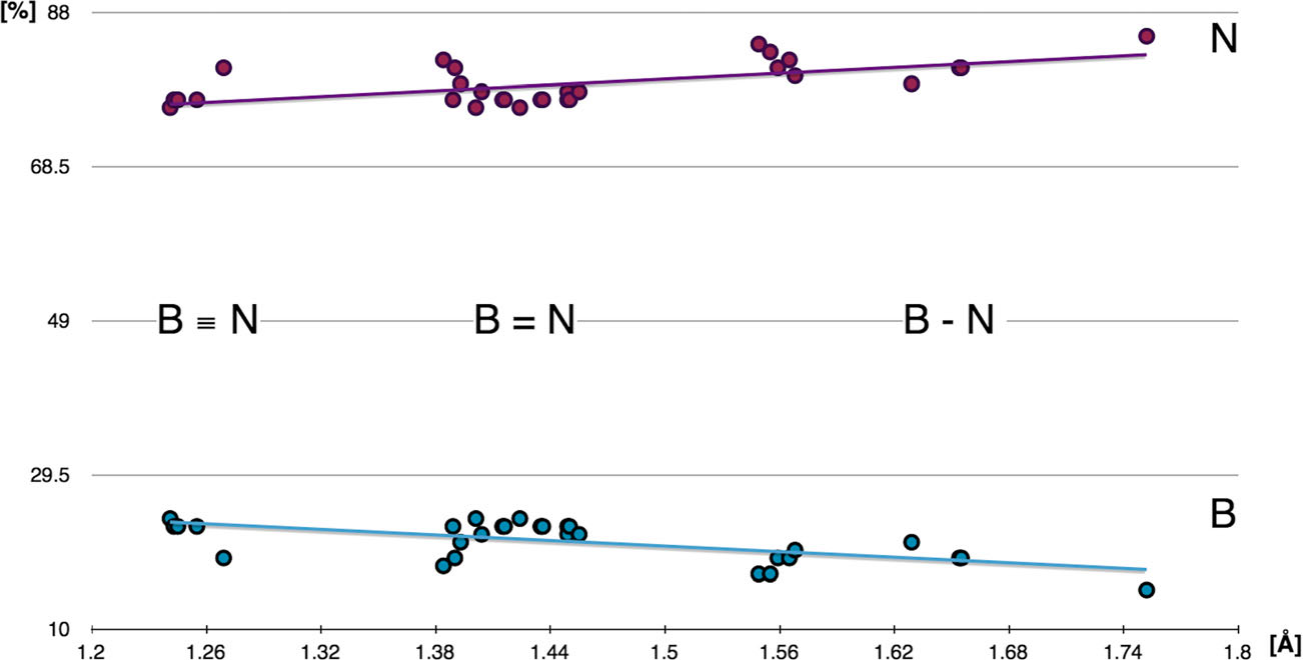
Correlation between percentage contributions of natural atomic hybrids at the B and N atoms, forming 2-center B-N bonds, and optimized bond length *r*_opt_(B,N) obtained from linear regression

The NBO analysis show that the percentage of the natural hybrid orbitals on the B and N atoms decreases and increases with elongation of the B≡N, B-N, and B-N bonds. The relationships studied using the linear regression model are shown in Fig. 15. Longer B-N bond is characterized by larger contributions of the NHOs on the N atom. This result stays in agreement with general picture of the BN bond dissociation showing that elongation of the bond leaves the lone pair on N atom.

## Conclusions

Application of the topological analysis of ELF enabled a detailed description and understanding of the local nature for 37 boron-nitrogen bonds with optimized lengths between 1.241 and 1.785 Å. A total of 25 molecules selected from the CSD have been investigated as well as prototypical H_3_N-BH_3_ molecule with a single dative bond (N→B) and the borazine molecule with the delocalized B^...^N bond. The BN bonds have been characterized with the wave function approximated within the DFT formalism using the M062X electron density functional in the real and Hilbert space using the topological analysis of η(*r*) and ρ(*r*) fields and the NBO analysis.

1. From the perspective of topological analysis of ELF, all investigated boron-nitrogen bonds are described by bonding disynaptic attractors, V(B,N). Thus, they have some covalent-polarized character.
2. The values of $\overline{N}$ between 5.57 and 5.72e support the classical concept of the triple B≡N bond, those between 3.50 are 4.54e support the double B=N bond, and those between 1.83 and 2.65e support a single B-N bond. However, there are also some BN bonds, not easily conforming to standard bonding, with populations between 2.90 and 3.15e, for which resonance forms of the B-N, B=N should be used. In summary, the results of topological analysis of ELF in general stay in agreement with the classical concept of the multiple BN bonds.
3. With the elongation of the BN bond, *r*_opt_(B,N), the population of the V(B,N) localization basin decreases from 5.72e, found for the triple B≡N bond (cetsup), to 1.83e obtained for the single B-N bond (cofvuo). Such results are in agreement with the power regression model.
4. The BN bonds are highly polarized towards the N atom. Majority of the electron density of the V(B,N) basin, between 91 and 96%, comes from the N quantum atom. This result could indicate formal dative (N→B) nature of the BN bonding. Polarity indices, *p*_NB_, are in the range from 0.77 to 0.93.
5. With elongation of the BN bond, contribution of the electron density from the N quantum atom decreases. At the same time, a very small decrease of electron density from the B quantum atom is observed.
6. The study performed using topological analysis of ρ(*r*) field shows typical features of the shared (ρ_(3,−1)_(*r*) > 0.09 e/au^3^, H_(3,−1)_(*r*) < 0) and closed-shell (∇^2^ρ_(3,−1)_(*r*) > 0) interactions.
7. The relationships between the ρ_(3,−1)_(*r*), H_(3,−1)_(*r*), and ∇^2^ρ_(3,−1)_(*r*) values and the *r*_opt_(B,N) bond length have been found and modeled using regression analysis. No relationship between the bond length and the ellipticity parameter, ε_(3,−1)_(*r*) have been observed. The power regression model explains in the best way the relationship between the DI values and the *r*_opt_(B,N) length.
8. The most interesting results are the values of the DI, describing exchange of electrons between quantum atoms, smaller than 1.28, even for very short, triple B≡N bonds. This finding is in contradiction with the values calculated for the triple bonds such as N≡N and HC≡CH, and emphasizes a difference between the BN bonds and other covalent bonds with shared electron density, despite the fact that all of them are formally classified as triple bonds.
9. The relationship between the basin population of V(B,N) and the delocalization index for the quantum atoms B and N has been described by a simple linear regression model. The model yields the population of 1.26e of V(B,N) for DI of 0. Such small population can be associated with electron density forming the lone pair on the N atom in molecules with long *r*_opt_(B,N) distance without covalent interactions.
10. The three NBOs of σ and π types confirmed the existence of 2-center natural bond orbitals in the molecules with formal triple B≡N bond. For the molecules with formal double B=N bonds, two-center σ and π NBOs have been observed for some molecules, whereas for other molecules, only single σ bonding orbital has been localized, accompanied by a lone pair orbital on the N atom. Similarly, for molecules with a single B-N bond, a single natural bond orbital has been found only for some of the studied molecules.
11. The NBOs, characterizing B-N, B=N, and B≡N bonds, are formed mainly by the natural atomic hybrid orbitals, centered on the N atom. The percentage contribution of the nitrogen atom ranges between 76 and 85% (σ, π), thus the character of the boron-nitrogen bonding, dominated by atomic orbitals of the N atom has been confirmed.
12. It is worth stressing that topological analysis of ELF and NBO analysis yields similar description of the BN bond. Both approaches show significant contribution of the nitrogen atom to the BN bonding, which is in agreement with a formal dative mechanism of its formation.
13. Percentage contributions of the atomic NHOs at the B and N atoms, forming molecular orbitals of the BN bonds, show small increase of the NHO at the N atom and a decrease of the NHO at the B atom, going from the shortest B≡N bonds to the longest B-N bonds.

## Electronic supplementary material

Figure S1. Optimised geometrical structure of the iditas molecule with values of the bond lengths and corresponding basin populations noted. (PNG 1338 kb)

High resolution image (TIFF 128 kb)

Figure S2. Correlation between the BN bond polarity, pNB, calculated using combined topological analysis of η(r) and ρ(r) functions and the optimised bond length ropt(B,N). (PNG 152 kb)

High resolution image (TIFF 223 kb)

Table S1. The experimental, rexp(B,N), and optimised bond lengths ropt(B,N) (in Å), obtained using different electron density functionals for four molecules with formal triple boronnitrogen bond. (DOC 29 kb)

## References

[1] Auwärter W (2019). Hexagonal boron nitride monolayers on metal supports: versatile templates for atoms, molecules and nanostructures. Sur Sci Rep.

[2] Wua W, Leng J, Mei H, Yang S (2018). Defect-rich, boron-nitrogen bonds-free and dual-doped graphenes for highly efficient oxygen reduction reaction. J Coll Inter Scien.

[3] Sheepwash E, Krampl V, Scopelliti R, Sereda O, Neels A, Severin K (2011). Molecular networks based on dative boron-nitrogen bonds. Angew Chem Int Ed.

[4] Icli B, Sheepwash E, Riis-Johannessen T, Schenk K, Filinchuk Y, Scopellitia R, Severin K (2011). Dative boron-nitrogen bonds in structural supramolecular chemistry: multicomponent assembly of prismatic organic cages. Chem Sci.

[5] Légaré M-A, Rang M, Bélanger-Chabot G, Schweizer J, Krummenacher I, Bertermann R, Arrowsmith M, Holthausen M, Braunschweig H (2019). The reductive coupling of dinitrogen. Science.

[6] Légaré M-A, Bélanger-Chabot G, Dewhurst RD, Welz E, Krummenacher I, Engels B, Braunschweig H (2018). Nitrogen fixation and reduction at boron. Science.

[7] Zhang Ya DH, Ma Y, Ji L, Guo H, Tian Z, Chen H, Huang H, Cui G, Asiri AM, Qu F, Chen L, Sun X (2019). Hexagonal boron nitride nanosheet for effective ambient N2 fixation to NH3. Nano Res.

[8] Saint-Louis CJ, Magill LL, Wilson JA, Schroeder AR, Harrell SE, Jackson NS, Trindell JA, Kim S, Fisch AR, Munro L, Catalano VJ, Webster CE, Vaughan PP, Molek KS, Schrock AK, Huggins MT (2016) The synthesis and characterization of highly fluorescent polycyclic azaborine chromophores. J Org Chem 81:10955–10963 10.1021/acs.joc.6b01998 27704820

[9] Lewis GN (1916). The Atom and the molecule. J Am Chem Soc.

[10] Becke AD, Edgecombe KE (1990). A simple measure of electron localization in atomic and molecular systems. J Chem Phys.

[11] Silvi B, Savin A (1994). Classification of chemical bonds based on topological analysis of electron localization functions. Nature.

[12] Savin A, Silvi B, Colonna F (1996). Topological analysis of the electron localization function applied to delocalized bonds. Can J Chem.

[13] Silvi B (2002). The synaptic order: a key concept to understand multicenter bonding. J Mol Struct.

[14] Chevreau H, Fuster F, Silvi B (2001). La liaison chimique: mythe ou réalité. Les méthodes topologiques de description de la liaison. L’Actualité Chimique.

[15] Savin A (2005). The electron localization function (ELF) and its relatives: interpretations and difficulties. J Mol Struct Theochem.

[16] Malcolm NOJ, Popelier PLA (2003). The full topology of the Laplacian of the electron density: scrutinising a physical basis for the VSEPR model. Faraday Discuss.

[17] The Cambridge Structural Database (CSD), https://www.ccdc.cam.ac.uk/solutions/csd-system/components/csd- accessed 23.11.2017

[18] Bader R (1994) Atoms in molecules: a quantum theory. Oxford University Press. USA.

[19] Weinhold F, Landis C (2005). Valency and bonding. A natural bond orbital donor-acceptor perspective.

[20] McNaught AD, Wilkinson A (compiled by) (1997) IUPAC. Compendium of chemical terminology. The “Gold Book”, 2nd ed. Blackwell Scientific Publications, Oxford. Online version (2019-) created by S. J Chalk. 10.1351/goldbook

[21] Mierzwa G, Gordon AJ, Berski S (2019). Topological analysis of electron localisation function: unlocking the nature of BC chemical bond. Possible existence of multiple bonds B=C and B≡C. Polyhedron.

[22] Mierzwa G, Gordon AJ, Latajka Z, Berski S (2015). On the multiple BO bonding using the topological analysis of electron localisation function (ELF). Comput Theor Chem.

[23] Berski S, Latajka Z, Gordon AJ (2011). On the multiple B–N bonding in boron compounds using the topological analysis of electron localization function (ELF). New J Chem.

[24] Mierzwa G, Gordon AJ, Berski S (2018). The electronic structure of molecules with the BF and BCl bond in light of the topological analysis of electron localization function: possibility of multiple bonds?. Int J Quantum Chem.

[25] Mierzwa G, Gordon AJ, Berski S (2018). On the nature of the boron–copper interaction. Topological study of the electron localisation function (ELF). New J Chem.

[26] Frisch MJ (2013). Gaussian 09 Revision E.01.

[27] Becke AD (1993). Density functional thermochemistry. III The role of exact exchange. J Chem Phys.

[28] Lee C, Yang W, Parr RG (1988). Development of the Colle-Salvetti correlation-energy formula into a functional of the electron density. Phys Rev.

[29] Vosko SH, Wilk L, Nusair M (1980). Accurate spin-dependent electron liquid correlation energies for local spin density calculations: a critical analysis. Can J Phys.

[30] Yan Z, Truhlar DG (2008). The M06 suite of density functionals for main group thermochemistry, thermochemical kinetics, noncovalent interactions, excited states, and transition elements: two new functionals and systematic testing of four M06-class functionals and 12 other functionals. Theor Chem Account.

[31] Chai J-D, Head-Gordon M (2008). Long-range corrected hybrid density functionals with damped atom–atom dispersion corrections. Phys Chem Chem Phys.

[32] Grimme S, Antony J, Ehrlich S, Krieg H (2010). A consistent and accurate ab initio parameterization of density functional dispersion correction (DFT-D) for the 94 elements H-Pu. J Chem Phys.

[33] Grimme S, Ehrlich S, Goerigk L (2011). Effect of the damping function in dispersion corrected density functional theory. J Comp Chem.

[34] Krishnan R, Binkley JS, Seeger R, Pople JA (1980). Self-consistent molecular orbital methods. XX A basis set for correlated wave functions. J Chem Phys.

[35] Clark T, Chandrasekhar J, Spitznagel GW, Von Ragué Schleyer P (1983). Efficient diffuse function-augmented basis sets for anion calculations. III. The 3-21+G basis set for first-row elements, Li-F. J Comput Chem.

[36] McLean AD, Chandler GS (1980). Contracted Gaussian basis sets for molecular calculations. I. Second row atoms, Z=11-18. J Chem Phys.

[37] Dunning TH (1989). Gaussian basis sets for use in correlated molecular calculations. I The atoms boron through neon and hydrogen. J Chem Phys.

[38] Kendall RA, Dunning TH, Harrison RJ (1992). Electron affinities of the first-row atoms revisited. Systematic basis sets and wave functions. J Chem Phys.

[39] Braunschweig H, Kupfer T, Radacki K, Schneider A, Seeler F, Uttinger K, Wu H (2008). Synthesis and reactivity studies of iminoboryl complexes. J Am Chem Soc.

[40] Frankhauser P, Pritzkow H, Siebert W (1994). Synthese und Struktur von l,2-Bis(organozinn-boryl)ethen-Derivaten / Synthesis and Structure of l,2-Bis(organotin-boryl)ethene Derivatives. Z Naturforsch B Chem Sci.

[41] Weigend F, Ahlrichs R (2005). Balanced basis sets of split valence, triple zeta valence and quadruple zeta valence quality for H to Rn: design and assessment of accuracy. Phys Chem Chem Phys.

[42] Andrae D, Häußermann U, Dolg M, Stoll H, Preuß H (1990). Energy-adjusted ab initio pseudopotentials for the second and third row transition elements. Theor Chim Acta.

[43] Campos CT, Jorge FE (2013). Triple zeta quality basis sets for atoms Rb through Xe: application in CCSD(T) atomic and molecular property calculations. Mol Phys.

[44] Feller D (1996). The role of databases in support of computational chemistry calculations. J Comp Chem.

[45] Pritchard BP, Altarawy D, Didier B, Gibson TD, Windus TL (2019). A new basis set exchange: an open, up-to-date resource for the molecular sciences community. J Chem Inf Model.

[46] Paetzold P, von Plotho C, Schmid G, Boese R, Schrader B, Bougeard D, Pfeiffer U, Gleiter R, Schafer W (1984). Darstellung, Reaktionen und Struktur vontert-Butyl(tert-butylimino)boran. Chem Ber.

[47] Paetzold P, von Plotho C, Schmid G, Boese R (1984). 1,3,5-Tri-tert-butyl-2,4,6-triisopropyl-3,5-diaza-1-azonia-2,6-dibora- 4-borata[2.2.0]bicyclohexan, der erste Vertreter der Dewarborazine/ 1,3,5-Tri-tert-butyl-2,4,6-triisopropyl-3,5-diaza-1-azonia-2,6-dibora- 4-borata[2.2.0]bicyclohexane, the First Dewar Borazine. Z Naturforsch B Chem Sci.

[48] Ziegler ML, Weidenhammer K, Autenrieth K, Friebolin H (1978) Die Molekül- und Kristallstrukturen von Methyl(4-Br-phenyl)amino-chlorphenylboran, (BrC_6_H_4_) (CH_3_)NB(C1)(C_6_H_5_), und Methyl(2-methyl-4-Br-phenyl)amino-chlor(2-methylphenyl)boran, (BrC_7_H_6_)(CH_3_)NB(Cl)(C_7_H_7_). Z Naturforsch B Chem Sci 33:200–208

[49] Noury S, Krokidis X, Fuster F, Silvi B (1997) TopMod Package, Paris

[50] Noury S, Krokidis X, Fuster F, Silvi B (1999). Computational tools for the electron localization function topological analysis. Comput Chem.

[51] Keith TA (2017) AIMAll (Version 17.01.25)

[52] Humphrey W, Dalke A, Schulten K (1996). VMD: visual molecular dynamics. J Mol Graph.

[53] Pettersen EF, Goddard TD, Huang CC, Couch GS, Greenblatt DM, Meng EC, Ferrin TE (2004). UCSF Chimera--a visualization system for exploratory research and analysis. J Comput Chem.

[54] Frisch MJ (2016). Gaussian 16, Revision E.01.

[55] Haase M, Klingebiel U, Boese R, Polk M (1986). Stabilisation and reactions of iminoboranes: crystal structure of (tert-butylimino)[tris(trimethylsilyl) silyl] borane. Chem Ber.

[56] Lermontova EK, Huang MM, Karlov SS, Zabalov MV, Churakov AV, Neumuller B, Zaitseva GS (2008). Synthesis and structures of new 1,3,6,2-dioxazaborocanes containing substituents in the ocane fragment. Russ Chem Bull.

[57] Elter G, Neuhaus M, Meller A, Schmidt-Base D (1990). 2,4,6-Tri-t-butylphenyl-substituierte iminoborane. J Organomet Chem.

[58] Rivard E, Merrill WA, Fettinger JC, Wolf R, Spikes GH, Power PP (2007). Boron−pnictogen multiple bonds: donor-stabilized P=B and As=B bonds and a hindered iminoborane with a B≡N triple bond. Inorg Chem.

[59] Silvi B, Fourré I, Alikhani M (2005). The topological analysis of the electron localization function. A key for a position space representation of chemical bonds. Monatsh Chem.

[60] Raub S, Jansen G (2001) A quantitative measure of bond polarity from the electron localization function and the theory of atoms in molecules. Theor Chem Accounts 106:223–232

[61] Bader RFW, Johnson S, Tang T-H, Popelier PLA (1996) The Electron Pair. J Phys Chem A 100:15398–15415

[62] Bader RFW, Heard GL (1999). The mapping of the conditional pair density onto the electron density. J Chem Phys.

[63] Cremer D, Kraka E (1984). A description of the chemical bond in terms of local properties of electron density and energy. Croat Chem Acta.

[64] Cremer D, Kraka E (1984). Chemical bonds without bonding electron density – does the difference electron-density analysis suffice for a description of the chemical bond?. Angew Chem.

[65] Bader Matta CF, Cortés-Guzmán F (2004). Where to draw the line in defining a molecular structure. Organometallics.

[66] Bader RFW (2009) Bond paths are not chemical bonds. J Phys Chem A 113:10391–10396 10.1021/jp906341r 19722600

[67] Mebs S, Beckmann J (2017) Real-space bonding indicator analysis of the donor acceptor complexes X_3_BNY_3_, X_3_AlNY_3_, X_3_BPY_3_, X_3_AlPY_3_ (X, Y = H, Me, Cl). J Phys Chem A 121:7717–7725 10.1021/acs.jpca.7b06977 28915044

[68] Weinhold F, Landis C (2005). Valency and bonding. A natural bond orbital donor-acceptor perspective.

[69] http://www.ccl.net/cca/software/NT/mopac6/nbo.htm, “NBO 3.0 Program Manual”, E. D. Glendening, A. E. Reed, J. E. Carpenter, F. Weinhold. accessed 11.06.2019.

[70] Braunschweig H, Homberger M, Hu C, Zheng X, Gullo E, Clentsmith G, Lutz M (2004) Synthesis and structure of [Cr{(η^6^-C_6_H_5_)2B{NtBu(SiMe_3_)}}] and [Cr{(η^6^-C_6_H_5_)_2_(BNMe_2_)_2_}], the first boron-bridged metalloarenophanes. Organometallics 23:1968–1970

[71] Müller P, Huck S, Köppel H, Pritzkow H, Siebert W (1995). Synhese und Strukturen von 9,10-Dihydro-9,10-diboraanthracen-Derivaten. Z Naturforsch B Chem Sci.

[72] Rodriguez MA, Borek TT (2013) 2,4-Bis(di­methyl­amino)-1,3,5-tri­methyl-6-(nitro­­oxy)borazine. Acta Crystallographica Section E: Structure Reports Online, 69: o634, accessed 23.11.2019. 10.1107/S1600536813007484 PMC3647835 23723801

[73] Robinson JMA, Kariuki BM, Philp D, Harris KDM (1997). Crystal engineering based on nitro derivatives of 10-hydroxy-10,9-borazarophenanthrene. Tetrahedron Lett.

[74] Hansch C, Leo A, Taft RW (1991). A survey of Hammett substituent constants and resonance and field parameters. Chem Rev.

[75] Yang X, Wu K, Yu Z (2015). BF3·OEt2-mediated alkenylation of pyrroles with α-oxo ketene dithioacetals. Tetrahedron Lett.

[76] Zheng X, Wang B, Herberich GE (2002) Borabenzene adducts of Ylidic Lewis bases. Syntheses and structures of 3,5-Me_2_C_5_H_3_BCH_2_PPh_3_, 3,5-Me_2_C_5_H_3_BCH(SiMe_3_)PPh_3_, and C_5_H_5_BN(Ph)PPh_3_. Organometallics 21:1949–1954

[77] Itoh K, Pkazaki K, Fujimoto M (2003). The structure of 1,3-enaminoketonatoboron difluorides in solution and in the solid state. Austr J Chem.

[78] Williams D, Pleune B, Kouvetakis J, Williams MD, Anderson RA (2000) Synthesis of LiBC_4_N_4_, BC_3_N_3_, and related C−N compounds of boron: new precursors to light element ceramics. J Am Chem Soc 122:7735–7741

[79] Kiviniemi S, Nissinen M, Alaviuhkola T, Rissanen K, Pursiainen J (2001). The complexation of tetraphenylborate with organic N-heteroaromatic cations. J Chem Soc Perkin Trans.

[80] Krokidis X, Noury S, Silvi B (1997). Characterization of elementary chemical processes by catastrophe theory. J Phys Chem A.

