# Supplementary figures and images for: The nature of multiple boron-nitrogen bonds studied using electron localization function (ELF), electron density (AIM), and natural bond orbital (NBO) methods
Source: J Mol Model. 2020 May 13;26(6):136. doi: 10.1007/s00894-020-04374-9 (PMC7220893; doi:10.1007/s00894-020-04374-9)

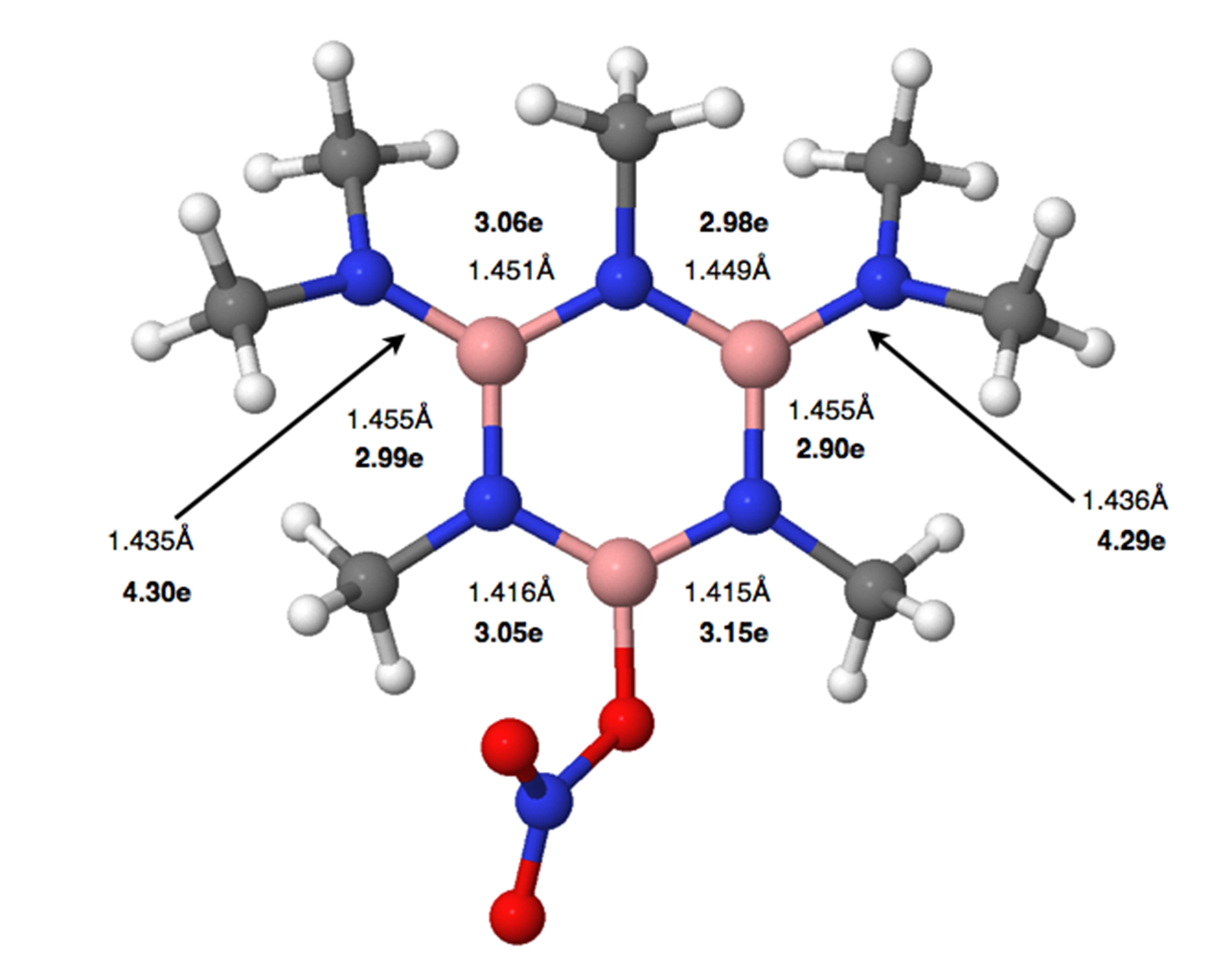

Supplement: Supplementary file 1 — Optimised geometrical structure of the iditas molecule with values of the bond lengths and corresponding basin populations noted. (PNG 1338 kb) [file 894_2020_4374_Fig16_ESM.png]

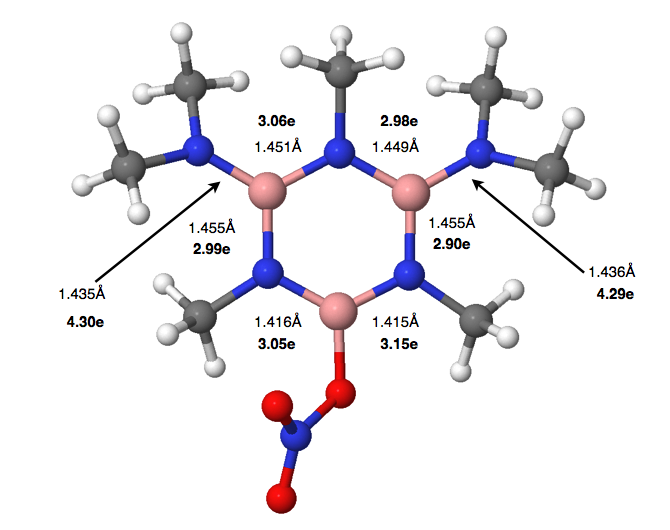

Supplement: Supplementary file 2 — High resolution image (TIFF 128 kb) [file 894_2020_4374_MOESM1_ESM.tiff]

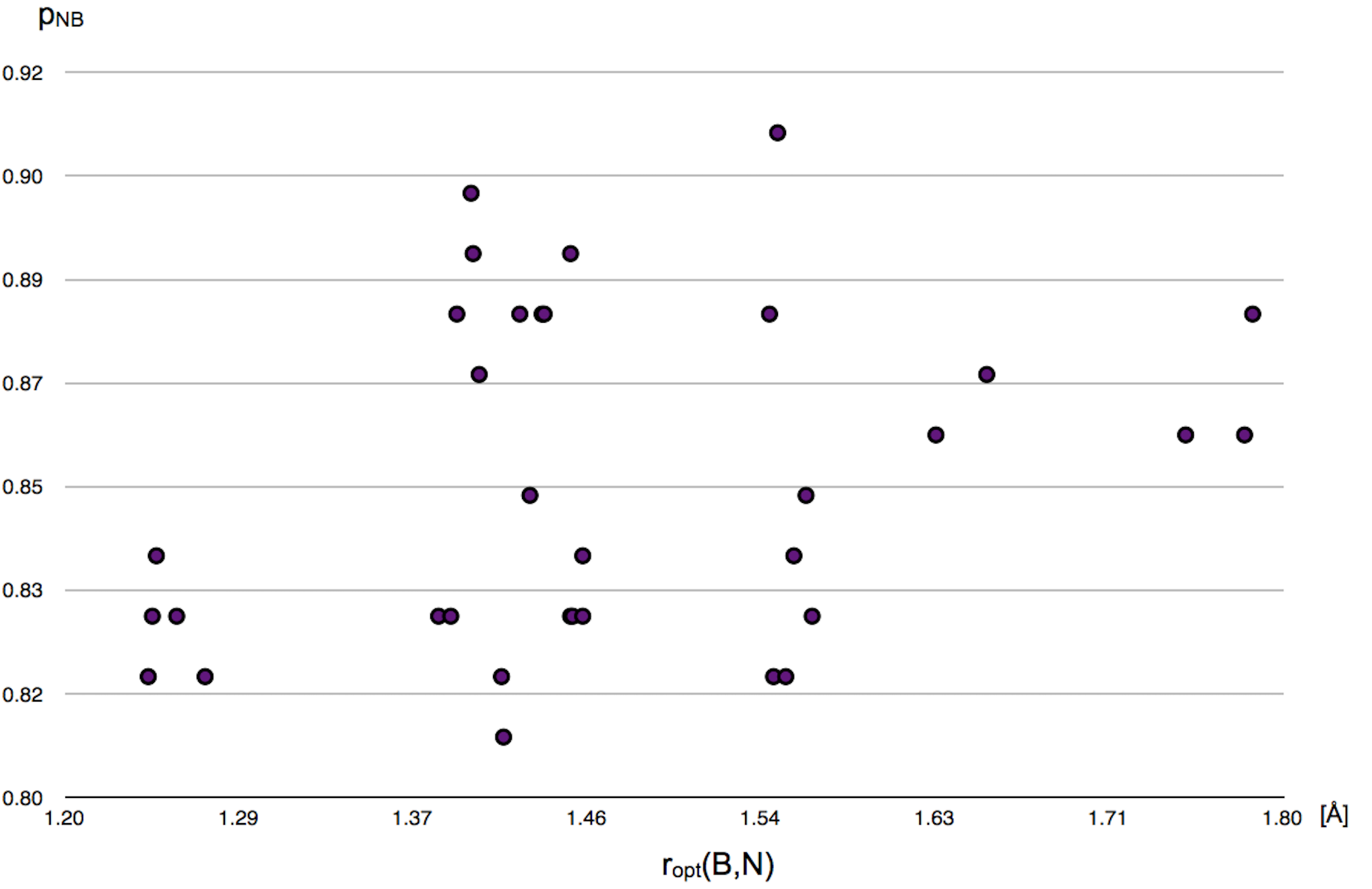

Supplement: Supplementary file 3 — Correlation between the BN bond polarity, pNB, calculated using combined topological analysis of η(r) and ρ(r) functions and the optimised bond length ropt(B,N). (PNG 152 kb) [file 894_2020_4374_Fig17_ESM.png]

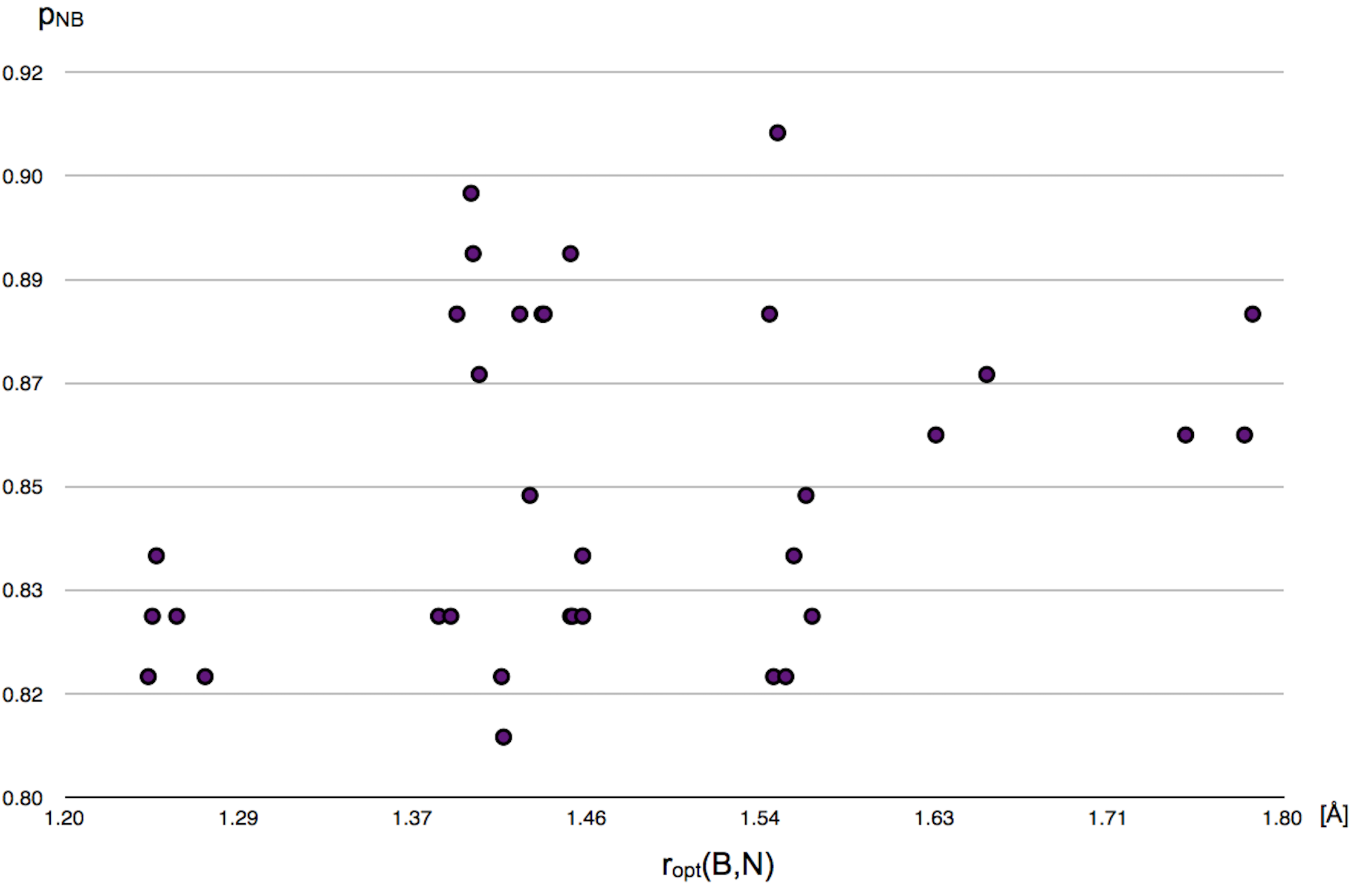

Supplement: Supplementary file 4 — High resolution image (TIFF 223 kb) [file 894_2020_4374_MOESM2_ESM.tiff]
