# Supplementary material for: The nature of multiple boron-nitrogen bonds studied using electron localization function (ELF), electron density (AIM), and natural bond orbital (NBO) methods
Source: J Mol Model. 2020 May 13;26(6):136. doi: 10.1007/s00894-020-04374-9 (PMC7220893; doi:10.1007/s00894-020-04374-9)
Supplement: Supplementary file 5 — The experimental, rexp(B,N), and optimised bond lengths ropt(B,N) (in Å), obtained using different electron density functionals for four molecules with formal triple boronnitrogen bond. (DOC 29 kb) [file 894_2020_4374_MOESM3_ESM.doc]

Table S1.

^1)^ single B-N bond,

Compoundrexp(B,N) M062XB3LYPB97D3ωB97X-Ddogpiy 1.2201.2451.2481.2571.244vejhib1.2321.2431.2471.2471.242sictii 1.2541.2551.2601.2601.254cetsup 1.2581.2411.2441.2441.241sictii 1)1.3741.3891.3921.3921.388
